# Thiolated Hyaluronic Acid: A Gateway for Targeted Killing of *Staphylococcus aureus* on the Race for Surface Colonization

**DOI:** 10.1002/adhm.202502890

**Published:** 2025-09-12

**Authors:** Mariana Blanco Massani, Susanne Meile, Annabelle Knoll, David Gintsburg, Ilaria Polidori, Anna Seybold, Débora C. Coraça‐Huber, Martin J. Loessner, Gergely Kali, Mathias Schmelcher, Szczepan Zapotoczny, Andreas Bernkop‐Schnürch

**Affiliations:** ^1^ Centre for Chemistry and Biomedicine (CCB) Department of Pharmaceutical Technology Institute of Pharmacy University of Innsbruck Innrain 80/82 Innsbruck 6020 Austria; ^2^ Institute of Food, Nutrition and Health ETH Zürich Schmelzbergstrasse 7 Zürich 8092 Switzerland; ^3^ Present address: Department of Immunology and Microbiology Anschutz Medical Campus University of Colorado Aurora CO 80045 USA; ^4^ Department of Zoology University of Innsbruck Innsbruck 6020 Austria; ^5^ Biofilm Lab, Experimental Orthopedics University Hospital for Orthopaedics and Traumatology Medical University Innsbruck Müllerstrasse 44, 1. Floor Innsbruck 6020 Austria; ^6^ Faculty of Chemistry Nanoengineering of Functional Polymeric Materials Group Jagiellonian University Gronostajowa 2 Krakow (Krakow‐Podgorze) 30–387 Poland; ^7^ Present address: Institute of Chemistry and Biotechnology Zurich University of Applied Sciences (ZHAW) Einsiedlerstrasse 31 8820 Wädenswil Switzerland

**Keywords:** enzyme‐responsive, phage endolysins, *Staphylococcus aureus*, targeted drug delivery, thiolated hyaluronic acid

## Abstract

Hyaluronic acid (HA) is degraded by Staphylococcal hyaluronate lyase (Hysa) and mammalian hyaluronidase (Hyal). Thiolated HA (HAMS) is used as a targeted gateway for *Staphylococcus aureus* killing while enhancing the previous M23 endolysin–polyphosphate (M23‐PP NPs) enzyme‐responsive nanoparticle formulation. Synthesis of HAMS and characterization for nuclear magnetic resonance, solubility, thiol content, pKa, and degradation by Hysa and Hyal are presented. Nanoparticles prepared via ionotropic gelation between M23‐PP NPs and either HAMS or HA yield M23‐PP/HAMS or M23‐PP/HA NPs, respectively. Their characterization includes size, zeta potential, morphology, release profiles, safety, targeted release, and efficacy. HAMS with a thiol content of 250.18 ± 90.32 µmol g^−1^, solubility of 50.99 ± 0.02 mg mL^−1^, exhibits pKa values of 3.2, 4.2, and 8.8. This thiolated polymer irreversibly inhibits Hyal activity, without affecting Hysa. M23‐PP/HAMS NPs (265 ± 47 nm, −25 mV) maintain their integrity for seven days at 37 °C, and HAMS coating prevents nonspecific degradation by Hyal, as confirmed by release studies. In a co‐culture ‘race for the surface’ experiment with MC3T3 osteoblasts and *S. aureus* ATCC 25923, M23‐PP/HAMS NPs produce 8‐log bacterial killing while promoting in vitro wound healing. These findings are pivotal to the development of new enzyme‐responsive excipients switchable by *S. aureus*.

## Graphical abstract

Thiolated hyaluronic acid (HAMS) synthesized and characterized by NMR, solubility, thiol content, and pKa, is degraded by Staphylococcal hyaluronate lyase but not by mammalian hyaluronidase. Coating polyphosphate–M23 phage endolysin nanoparticles (M23‐PP) with HAMS confers *Staphylococcus aureus* responsiveness. M23‐PP/HAMS are studied for size, charge, morphology, release profile, safety, and efficacy. M23‐PP/HAMS provides a switch‐response to avoid *S. aureus* implant‐related infection.

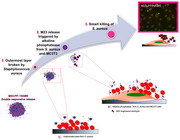

## 1 Introduction

Hyaluronic acid (HA), a vital component of the extracellular matrix, is a linear heteropolysaccharide composed of alternating residues of D‐glucuronic acid and N‐acetyl‐D‐glucosamine, linked by β−1,3 and β−1,4 glycosidic bonds, respectively.^[^ ^1^, ^2^, ^3^ ^]^ This polymer plays a crucial role in wound healing and tissue regeneration by regulating cell adhesion, migration, and proliferation while providing mechanical support and controlling tissue hydration.^[^ ^4^, ^5^, ^6^ ^]^

Hyaluronate lyase (Hysa) and hyaluronidase (Hyal), respectively found in bacteria and mammals, are enzymes that break down HA.^[^ ^3^ ^]^ They differ in structure, function, and the mechanism of reaction to degrade HA.^[^ ^7^, ^8^, ^9^ ^]^ Particularly, *Staphylococcus aureus* secretes Hysa as a spreading factor that cleaves HA during infection and biofilm dispersal.^[^ ^3^, ^10^, ^11^ ^]^ In humans, the turnover of hyaluronan by the Hyal enzyme family is critical for normal extracellular matrix remodeling.^[^ ^12^ ^]^

We have previously developed an enzyme‐responsive nanoparticleformulation (M23‐PP NPs) that was able to eradicate *S. aureus* biofilm in 2 h.^[^ ^13^ ^]^ However, these particles have two characteristics that can be significantly improved: i) the stability of this formulation was limited as the NPs aggregated over time; ii) the targeted release given by enzyme‐responsiveness could be compromised by the high alkaline phosphatase (AP) activity produced by mammalian cells. Enzyme‐responsive delivery systems can optimize drug bioavailability, releasing active pharmaceutical ingredients at the target site, maximizing therapeutic effect while reducing side effects associated with overdosage.^[^ ^14^, ^15^ ^]^ Previous studies on the modification of polysaccharides with mercaptosuccinic acid (MS) have primarily focused on mucoadhesive and permeation‐enhancing properties, without exploring their potential for nanoparticle (NP) stabilization.^[^ ^16^, ^17^ ^]^ We hypothesize that modifying HA with a biocompatible capping agent, such as MS,^[^ ^18^ ^]^ could enhance the stability of our M23‐PP NPs through the formation of a HAMS coating. Additionally, unlike bacterial Hysa, vertebrate Hyal contains thiol groups in its binding site,^[^ ^5^ ^]^ and there are some indications that the activity of Hyal is inhibited by thiol‐containing molecules.^[^ ^5^, ^19^ ^]^ So far, the degradation of thiolated hyaluronic acid by Hysa and its potential use as a coating for bacteria‐triggered release have not been characterized. We have, therefore, identified an opportunity within this gap in knowledge and hypothesized that Hysa and Hyal exhibit different cleavage efficiencies, making HAMS‐coated M23‐PP NPs (M23‐PP/HAMS) more specifically responsive to *S. aureus*.

To test our hypothesis, we aimed to synthesize HAMS using a well‐established method developed by our group.^[^ ^17^ ^]^ The obtained polymer was characterized by ^1^H‐NMR, thiol content, solubility, and pKa. Differential affinity between HA and HAMS used as substrates for bacterial Hysa and mammalian Hyal was studied. Ionotropic gelation was used to form HAMS or HA‐coated M23‐PP NPs, M23‐PP/HAMS, and M23‐PP/HA, respectively. The NPs were characterized by dynamic light scattering (DLS) and energy‐filtered transmission electron microscopy (EFTEM). Stability and the provision of an additional stimulus‐responsive barrier to the AP‐triggered switch were investigated by in vitro release experiments. Finally, safety and efficacy on ´the race for the surface´ between MC3T3 and *S. aureus* ATCC 25923 in co‐culture experiments were explored.

## 2 Results and Discussion

### 2.1 Synthesis of Hyaluronic Acid Derivative (HAMS)

Mercaptosuccinic acid (MS) is a biocompatible thiolated diacid previously studied for several medical applications. Particularly, it can be used as a capping agent in order to avoid the over‐growth of nanoparticles, preventing their aggregation/coagulation in colloidal synthesis.^[^ ^18^ ^]^ Moreover, the improvement of the drug delivery properties of polysaccharides by esterification of OH groups using S‐acetyl mercaptosuccinic anhydride was previously performed by our group.^[^ ^17^ ^]^

**Figure 1:**
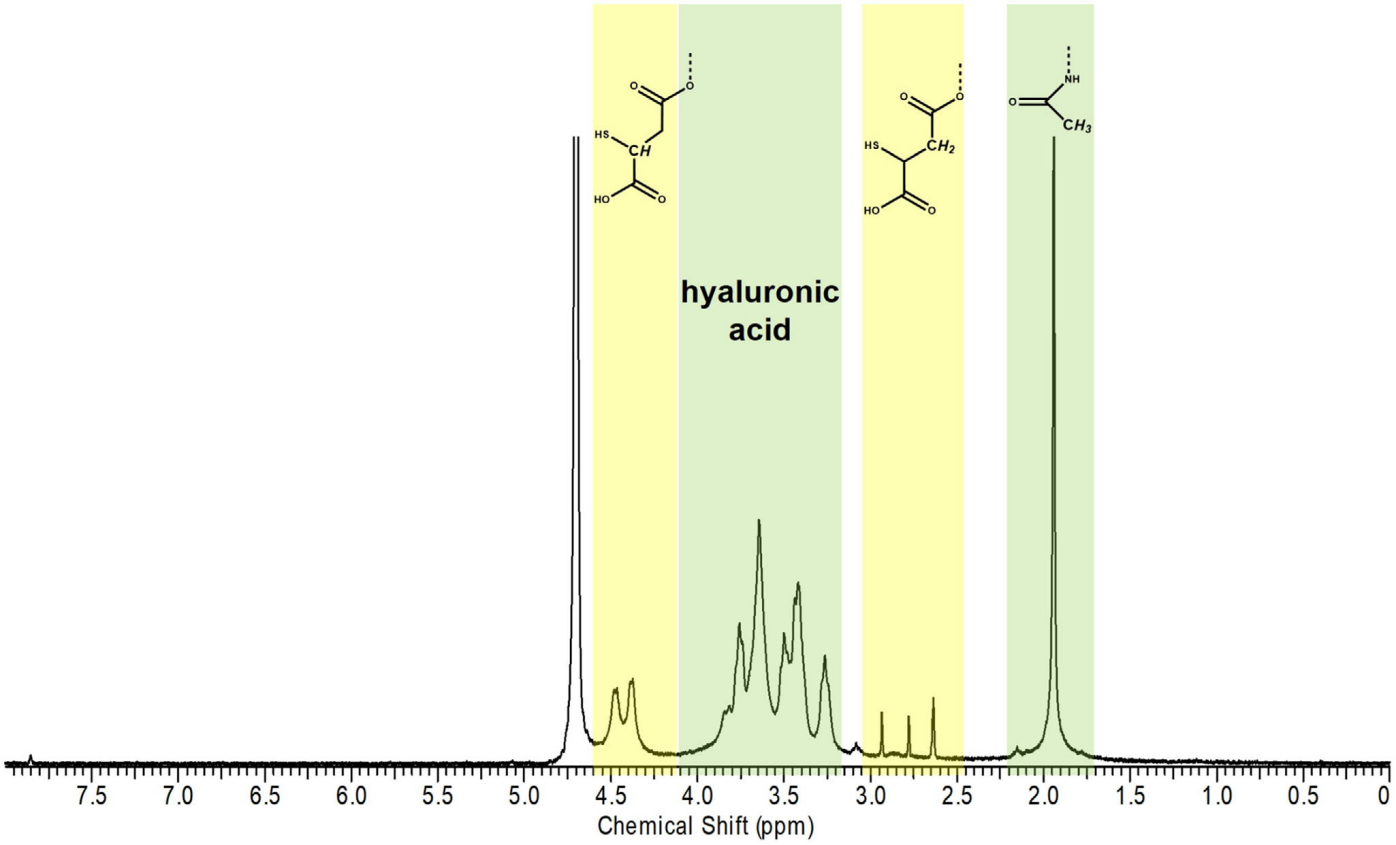
400 MHz ^1^H‐NMR spectrum of the synthesized hyaluronic acid mercaptosuccinate (HAMS) in D_2_O.

**Scheme 1:**
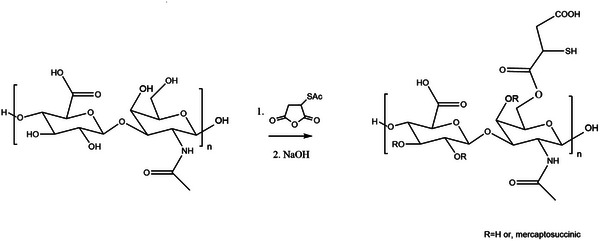
Scheme of reaction for obtaining hyaluronic acid mercaptosuccinate. The most likely product is shown in scheme 1, assuming esterification occurs at the C1 position; i.e., the carboxyl group adjacent to the thiol (─COOH).

After esterification, a treatment at alkaline conditions is suggested to deacetylate the S‐acetyl protecting group. However, under this condition, the ester group could also be hydrolyzed,^[^ ^20^ ^]^ lowering the thiol content. Therefore, we screened deprotection conditions to balance deacetylation with HA‐thiolation (Figure S1, Supporting Information). The ^1^H‐NMR spectrum of the product obtained after deacetylation is shown in **Figure** **1**. Besides the peaks of hyaluronic acid between 3.05 and 3.90 ppm, and the acetyl protons of the N‐acetyl‐D‐glucosamine repeats at 1.95 ppm, the methine and methylene signals of the conjugated mercaptosuccinic acid at the regions of 4.50–4.40 and 2.95–2.65 ppm, respectively, confirmed the success of the reaction. These peaks are in good agreement with previous mercaptosuccinic acid modified polysaccharides,^[^ ^16^, ^17^ ^]^ with some upfielded shifts due to the different chemical environment in hyaluronic acid. Determining the pKa of polymer excipients is essential for identifying conditions that minimize the repulsive potential between charged species during polyelectrolyte complexation.^[^ ^15^, ^21^ ^]^ Here, HAMS is intended to be used as a coating for M23‐PP NPs previously formulated at pH 3.^[^ ^13^ ^]^ From the distribution of species in the protonation‐deprotonation equilibria (Figure S2, Supporting Information), it can be predicted that esterification of HA mainly occurs at the C1 position (**Scheme** **1**); i.e., the carboxyl group adjacent to the thiol (─COOH), which has a pKa of ≈2.5–3.0, with the remaining carboxylic acid at the C4 position (the distal ─COOH, pKa ≈4.0–5.0)^[^ ^22^ ^]^ determining the polymer's properties. This suggests that formulating at a pH 3, which is below the pKa_2_ from HAMS (**Table** **1**), would be a reasonable condition to achieve interaction between negatively charged M23‐PP NPs and HAMS.

**Table 1:** Physicochemical parameters of HAMS.

pka_1_ ^a)^	pka_2_ ^a)^	pka_3_ ^a)^	Total SH^a)^ [µmol g^−1^]	S‐S^a)^ [µmol g^−1^]	Free SH^a)^ [µmol g^−1^]	Solubility^a)^ [g L^−1^]
3.2 ± 0.1	4.2 ± 0.1	8.8 ± 0.2	250.18 ± 90.32	70.07 ± 45.16	110.04 ± 18.61	50.99 ± 0.02

^a)^ Values are means of 3 independent replicates ± SD.

The degree of thiolation obtained after synthesis (Table 1) was in the range of that described previously for cellulose−mercaptosuccinate using the same method.^[^ ^17^ ^]^ Based on Ellman's assay and disulfide quantification, 250.18 ± 90.32 µmol g^−1^ of thiol groups were functionalized onto the hyaluronan backbone, corresponding to a degree of thiolation of 10.03 ± 3.6%, calculated relative to the repeating disaccharide units of hyaluronan. ^1^H NMR analysis validated these findings, yielding a similar degree of thiolation of 13.5%, determined by integrating the hyaluronan signals between 3.10 and 4.15 ppm (corresponding to 10 protons) and the methylene protons of MSA between 2.60 and 2.95 ppm (2 protons). The solubility in water was not changed after modification compared with HA (51.00 ± 0.01 g L^−1^). This high‐water solubility is promising as it allows the screening of a wide range of HAMS concentrations for achieving the coating of M23‐PP NPs, as will be further discussed (Section 2.3).

The absolute weight average molar masses ($\bar{M}$ *_w_*) of the precursor HA and HAMS were 21400 Da and 24200 Da, respectively. These values align with the degree of thiolation observed after the chemical modification of HA (Table 1).

### 2.2 Hysa and Hyal Enzymatic Cleavage of HAMS Compared to HA

**Figure 2:**
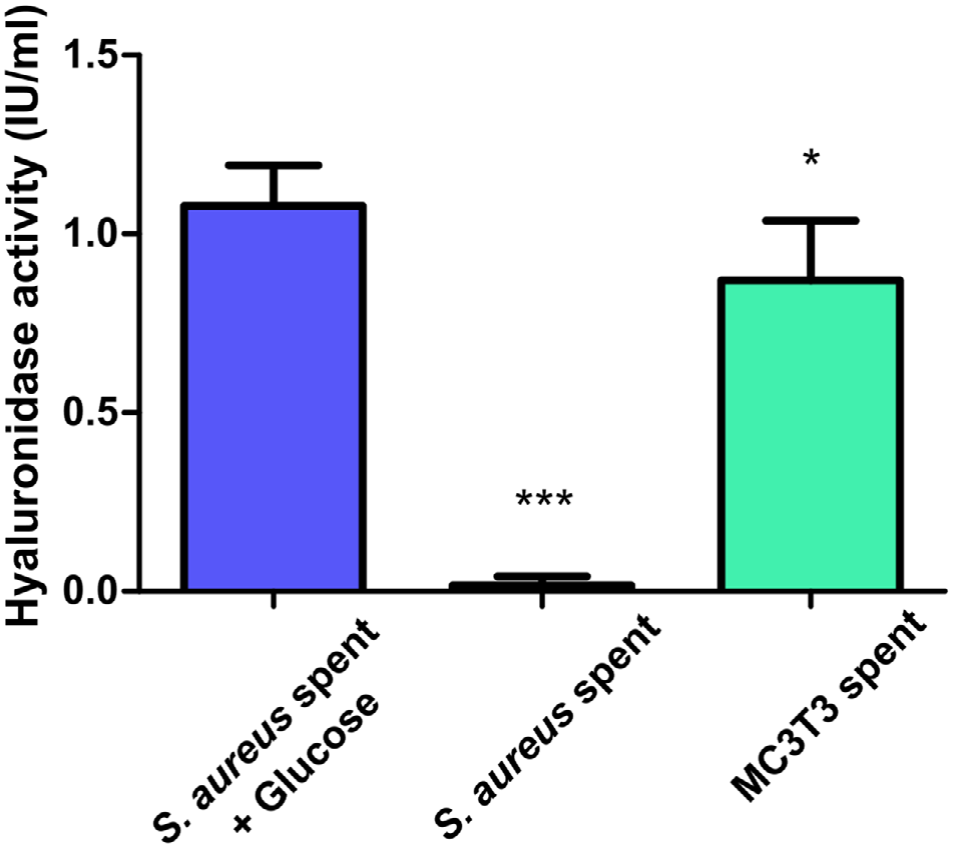
Hyal activity in the spent of *S. aureus* and MC3TC3 was estimated as HA depletion. BT‐Hyal was used as a reference enzyme for this determination. Values are means of 6 replicates ± SD. ^*^ *p*<0.05, ^***^ *p*<0.001.

Hysa and Hyal are collectively known as the enzymes that break down HA.^[^ ^3^ ^]^ These enzymes can be found in bacteria and mammals, respectively. They differ in structure, function, and the mechanism of reaction to degrade HA.^[^ ^7^, ^8^, ^9^ ^]^ Particularly, *Staphylococcus aureus* secretes Hysa as a spreading factor that cleaves HA during infection, and its production is correlated with biofilm dispersal.^[^ ^3^, ^10^, ^11^ ^]^ On the other hand, turnover of hyaluronan by the Hyal enzyme family is critical for normal extracellular matrix remodeling in humans.^[^ ^12^ ^]^ Furthermore, Hyal has been suggested to play a role in bone remodeling by extracellularly regulating the HA turnover in the bone environment.^[^ ^23^ ^]^ Here, we studied the ability of *S. aureus* and MC3T3 to deplete HA and correlated it to Hyal activity using BT‐Hyal as a reference for substrate depletion (**Figure** **2**). Only spent media from *S. aureus* ATCC 25923 supplemented with glucose was able to deplete HA (**Figure** 2). In line with previous publications, this suggests the involvement of Hysa in *S. aureus* ATCC 25923 biofilm cycle, as the latter condition is used to promote biofilm formation in this strain.^[^ ^13^ ^]^ Moreover, the activity exerted by *S. aureus* (1.08 ± 0.11 N = 6) was in the range of that observed by MC3T3 spent (0.87 ± 0.17 N = 6) (Figure 2).

**Figure 3:**
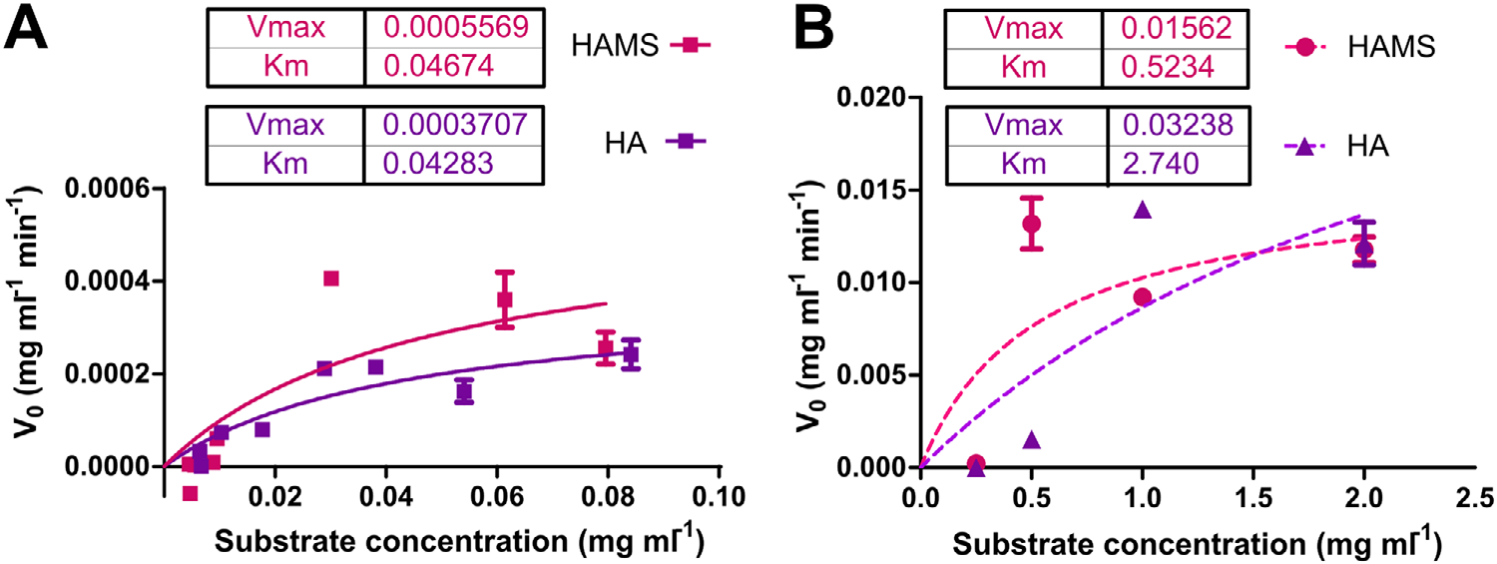
V_max_ and K_m_ estimation for 500 IU mL^−1^ of A) S‐Hysa, and B) BT‐Hyal when using HA (▪) and HAMS (▪) as substrates. Each point represents the mean of three replicates; error bars denote standard deviation (SD).

Target selectivity for a drug can be achieved based on differential sensitivity or accessibility.^[^ ^14^ ^]^ In order to assess the potential use of HAMS as a coating for targeted release triggered by bacteria, we checked the kinetics of degradation of HAMS and HA by bacterial S‐Hysa (**Figure** **3**A) and mammalian BT‐Hyal (Figure 3B). Both enzymes followed classic Michaelis–Menten kinetics at low substrate concentrations.^[^ ^7^ ^]^ Therefore, it could be assumed that a single substrate binds the active site of the enzyme, and the initial rate of degradation is proportional to the concentration of the enzyme–substrate complex.^[^ ^24^ ^]^ K_m_ of the bacterial S‐Hysa surrogate was in the same range for either HAMS or HA substrate (Figure 3A), indicating that Hyla shows similar affinity for both substrates.^[^ ^25^ ^]^ Surprisingly, the Michaelis constant K_m_ and V_max_ for BT‐Hyal using HA and HAMS as substrates (Figure 3B) decreased when the substrate was thiolated. This indicates higher affinity for HAMS and a slower degradation rate of this substrate, a behavior that was paired with HAMS acting as an irreversible inhibitor of BT‐Hyal, as confirmed in Figures S3–S5 (Supporting Information).

**Figure 4:**
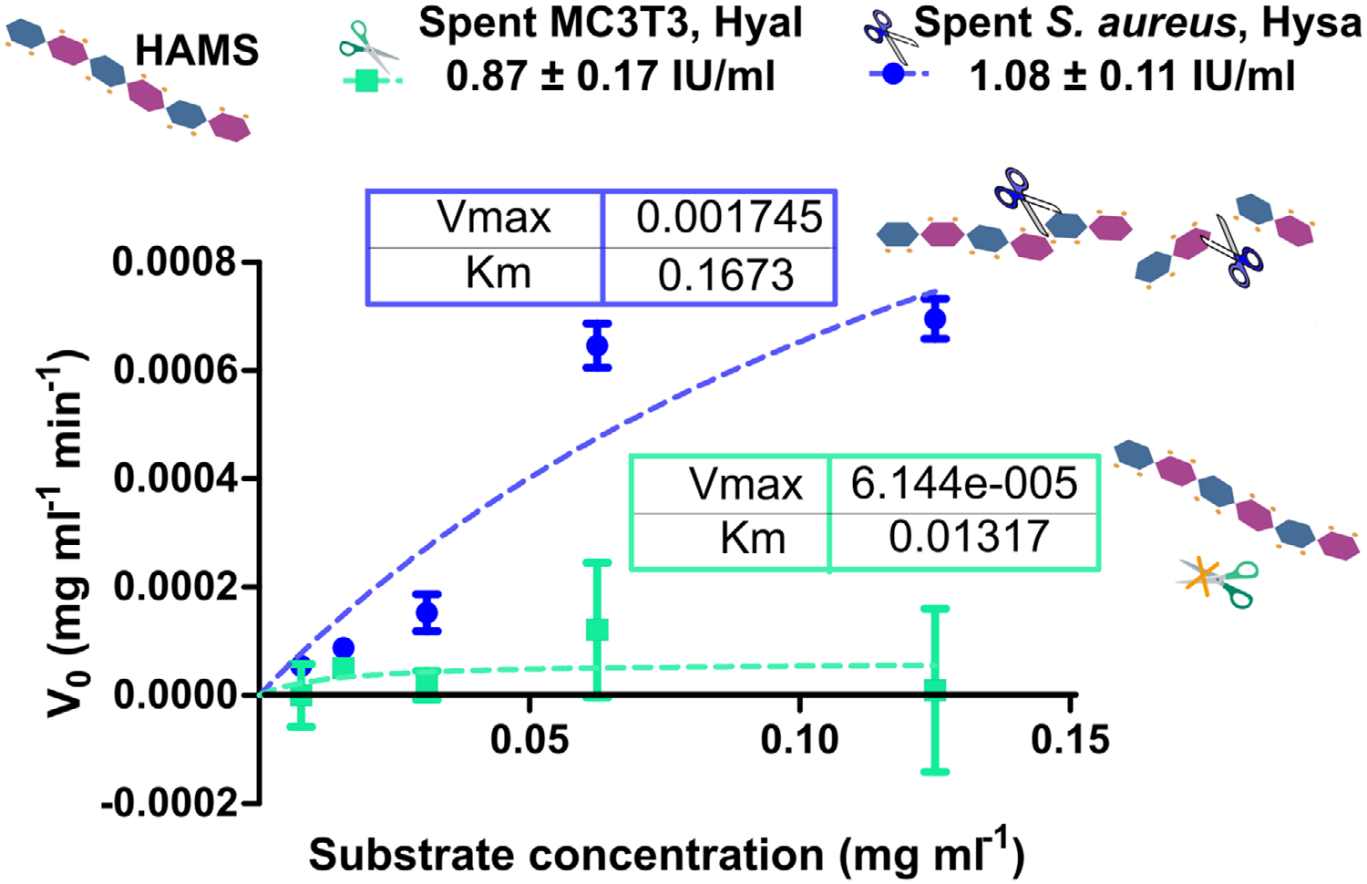
V_max_ and K_m_ estimation for the degradation of HAMS by spent MC3T3 and *S. aureus* ATCC 25923 containing equiactive concentrations of Hyal and Hysa, respectively. Each point represents the mean of three replicates; error bars denote standard deviation (SD).

Given the observed differential behavior of bacterial S‐Hysa and mammalian BT‐Hyal surrogates toward HAMS substrate, we wanted to assess the potential of this polymer excipient as a switch for targeted disruption by *S. aureus*. Therefore, we studied the substrate degradation produced by spent *S. aureus* ATCC 25923 and MC3T3 cells, confirming the ability of bacterial Hysa, but not mammalian Hyal, to degrade HAMS substrate (**Figure** **4**). This result shows the great potential of HAMS for developing enzyme‐responsive delivery systems targeting *S. aureus*. Therefore, as we will show in the following sections, HAMS was used as a coating for M23‐PP NPs previously optimized by Blanco Massani et al.^[^ ^13^ ^]^

### 2.3 Nanoparticles Formation and Characterization

**Figure 5:**
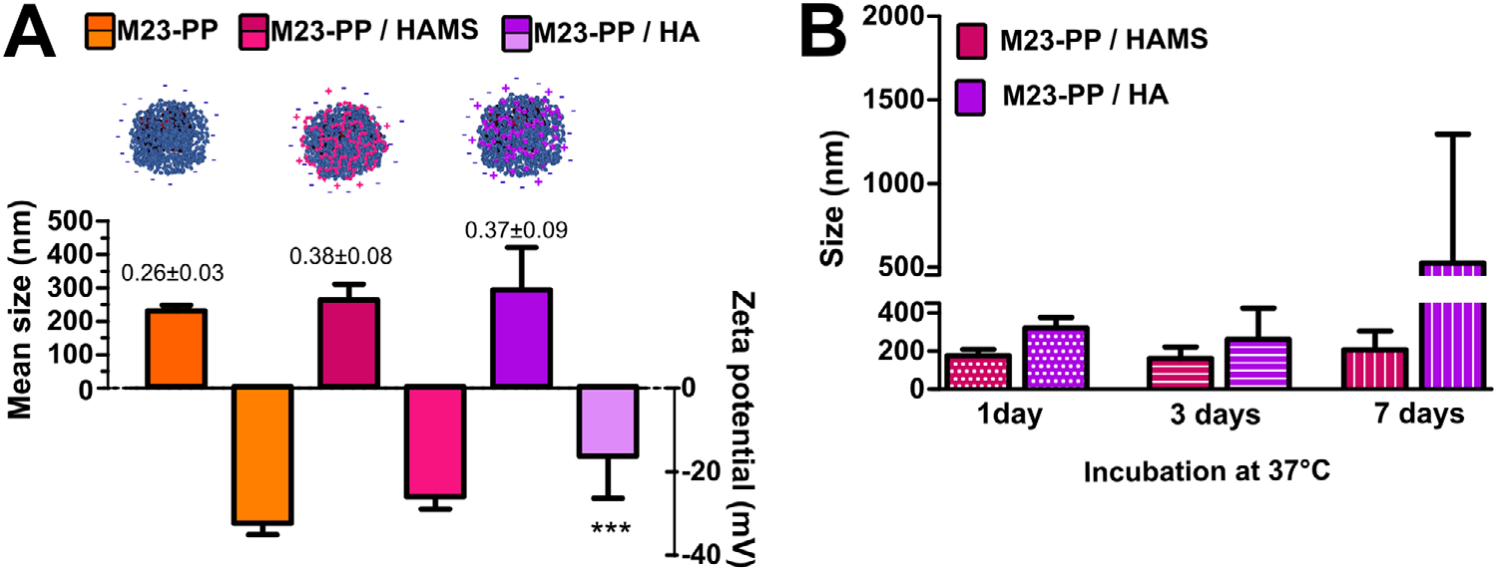
A) Mean size (nm) (bars), PDI (number above bars), and zeta potential (mV) obtained when M23‐PP NPs were coated with HAMS or HA. Particles are schematically represented by spheres not drawn to scale. B) Size variations of the indicated samples stored for different time periods. Values are means of at least 6 replicates ± SD.

The high solubility of HA and HAMS in water allowed us to screen a wide concentration of polymer to coat M23‐PP NPs (Supporting information: Screening_HA and HAMS coated PP‐M23 NPs.xls). Nanoparticles with the lower PDI were obtained when dropping M23‐PP NPs in 36.2 µm of HAMS or 40.9 µm of HA (**Figure** **5**A). A non‐significant shift in zeta potential was observed for M23‐PP NPs / HAMS, which maintained a charge of ≈−25 mV. This may be explained by the co‐existence of H_2_B^−^ and HB^2−^ in equilibrium (Figure S2, Supporting Information) at the final pH of the formulation (M23‐PP / HAMS pH 3.183). In contrast, HA coating produced a shift in charge to less negative values at a similar pH (−16 mV, M23‐PP / HA pH 3.156), in coincidence with the equilibrium hyaluronic acid/hyaluronate.^[^ ^26^ ^]^ The particle size visualized by TEM (Figures S6 and S7, Supporting Information) showed good agreement with the DLS data. Given that a concentration >20.7 µm of HAMS is expected to cause complete inhibition of the mammalian Hyal as described in the supporting information (Figure S3, Supporting Information), the HAMS‐coated particles obtained show great potential for achieving targeted release, as will be discussed in Section 2.4. MIC and MBC from M23‐PP NPs were 39<MIC≤156 nm in M23, while MAC was 39 nm, with no significant changes after coating with either HAMS or HA (see supporting information MIC, MBC, and MAC.xls and Figure S8, Supporting Information). These are in coincidence with our previous studies.^[^ ^13^ ^]^ Altogether, the results demonstrate that the spent medium from *S. aureus* ATCC 25923 is able to break down HAMS and HA coatings, leading to the release of M23‐PP NPs cargo. Once released, the cargo can be subsequently cleaved by AP,^[^ ^13^ ^]^ effectively killing the target and inhibiting further biofilm formation.

M23‐PP / HAMS maintained their integrity for 7 days, while M23‐PP / HA agglomerated after this period when stored at 37 °C (Figure 5B, Figure S9, Supporting Information). The HA‐based coating enhanced the bactericidal stability of M23‐PP over the storage period, as discussed in Table S1 (Supporting Information).

### 2.4 HAMS Protection Against Unspecific Release

**Figure 6:**
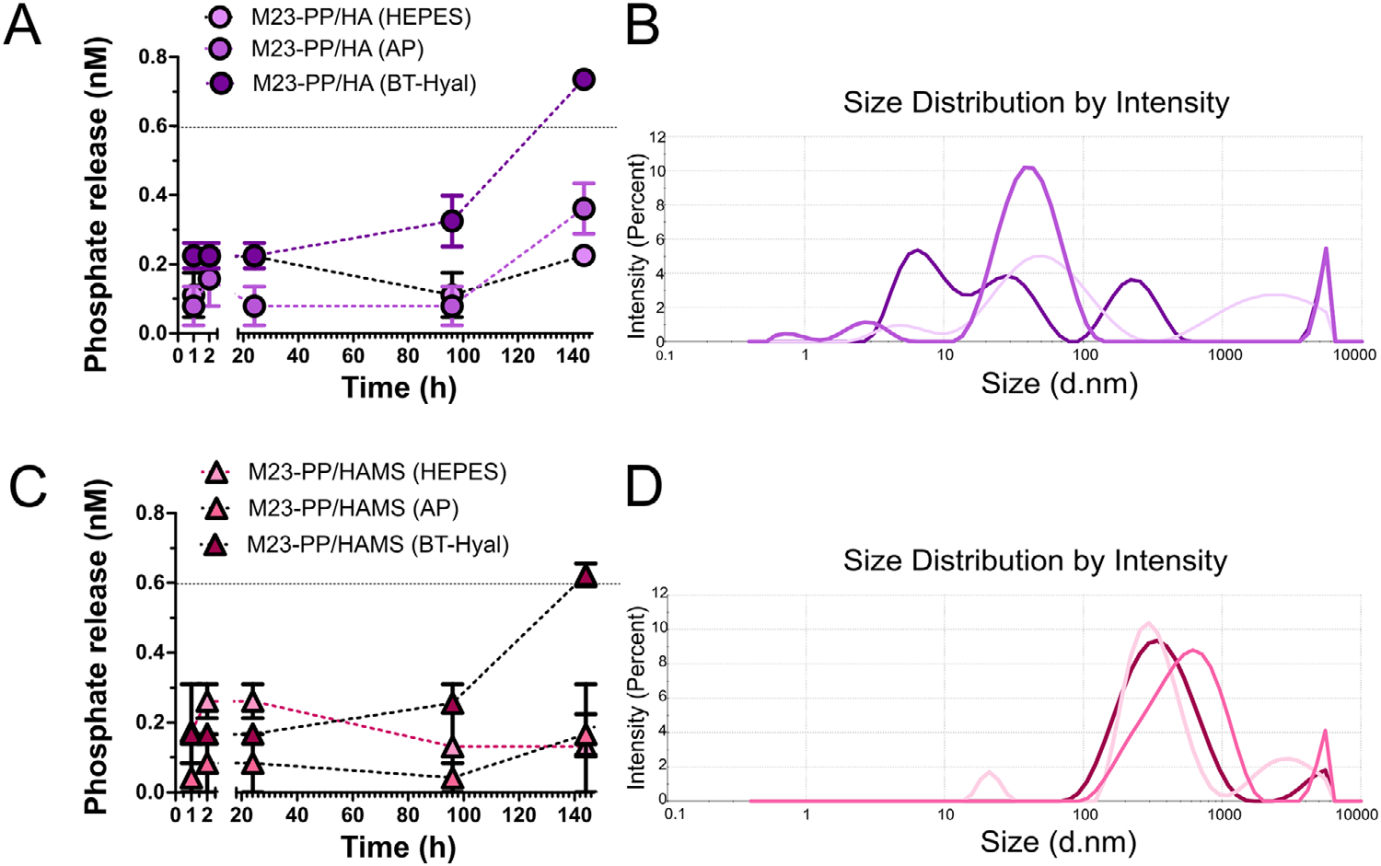
Cumulative phosphate release triggered by AP and BT‐Hyal for A) M23‐PP/HA NPs and C) M23‐PP/HAMS NPs compared to HEPES control. B) and D) are the respective particle size distributions at the end of the experiment. The dotted line at ≈0.6 nm in panels A and C is included to enhance clarity and highlight key data points. Each point represents the mean of four replicates; error bars denote standard deviation (SD).

To further validate the hypothesis of HAMS acting as an inhibitor of mammalian Hyal, we studied and compared the release of phosphate from M23‐PP NPs coated with HA and HAMS under different conditions. As depicted in **Figure** **6**, release of phosphate triggered by AP from M23‐PP / HA (Figure 6A) and M23‐PP / HAMS (Figure 6C) was not higher than the phosphate released in HEPES buffer. This confirms that the outermost coating of both particles was composed mostly of HA‐based materials. After 144 h of incubation with AP, M23‐PP / HA NPs released ca 0.4 nm of phosphate, while release was below 0.3 nm for M23‐PP / HAMS NPs. This result contrasts with M23‐PP NPs phosphate release under the same conditions,^[^ ^13^ ^]^ for which AP triggered the release of 400 nm of phosphate from M23‐PP NPs, already after 24 h. This confirms the protective effect of HA‐based coatings against the action of AP, which seems to be increased by the stability of HAMS‐coated particles (Figure 6D, in contrast to Figure 6B).

In the presence of BT‐Hyal, the release of phosphate was higher for M23‐PP / HA NPs than that for M23‐PP / HAMS, a result that was more noticeable after 144 h of incubation. Interestingly, M23‐PP / HA NPs particles lost their structure after 144 h of incubation under different conditions (Figure 6B), while size was not dramatically changed for M23‐PP / HAMS (Figure 6D). Additionally, the zeta potential of M23‐PP / HAMS was negative after the release experiments, which could be attributed to the neutral pH of HEPES buffer (HEPES: −16.47 ± 0.64, AP‐13.6 ± 0.6, Hyal: −10.77 ± 0.45). Altogether, this shows the potential of HAMS coating to avoid nonspecific release under the action of vertebrate Hyal. As the concentration of HAMS to obtain M23‐PP/HAMS NPs is 36.2 µm (discussed in Section 2.3), which is in the range of that causing total inhibition of BT‐Hyal (Figure S3, Supporting Information), the obtained NPs are promising for the targeted killing of *S. aureus*. Therefore, we proceeded to investigate the mechanism of action of M23‐PP / HAMS NPs to validate their targeted release capabilities (Section 2.5).

### 2.5 The Role of Hysa in M23‐PP/HAMS NPs Efficacy

**Figure 7:**
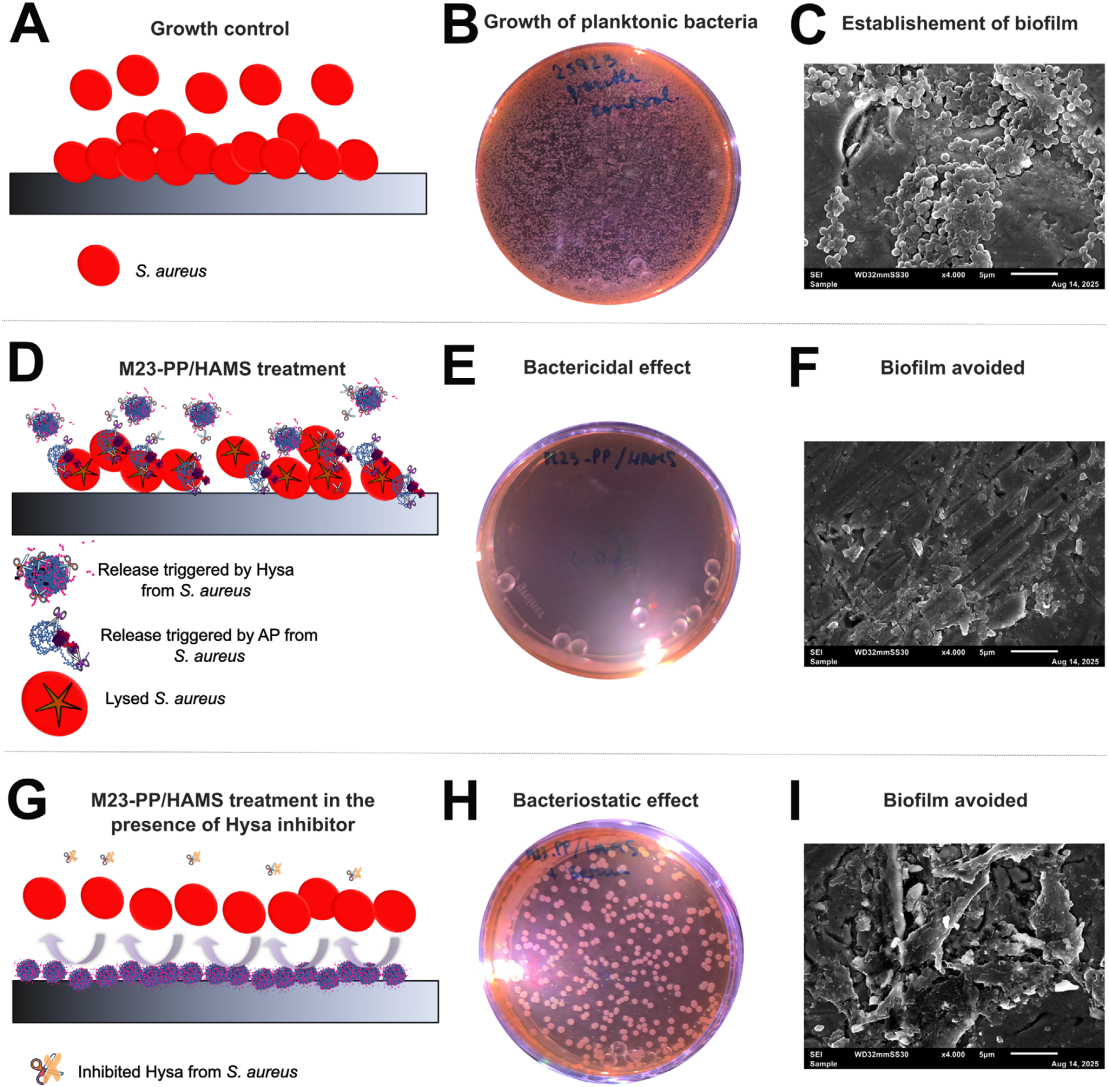
Behavior of *S. aureus* incubated in contact with an implant under conditions promoting biofilm formation^[^ ^13^ ^]^ (growth control, A–C), in the presence of M23‐PP/HAMS NPs (M23‐PP/HAMS treatment, D–F) and using serum as inhibitor of Hysa^[^ ^11^ ^]^ (M23‐PP/HAMS treatment in the presence of Hysa inhibitor, G–I). (A,D,G) Represent the schematic interpretation of results based on the (B,E,H) growth and (C,F,I) biofilm formation in the presence of the mentioned treatments.

To better understand the mechanism of action of M23‐PP/HAMS NPs and verify the Hysa‐triggered efficacy, we run a gating experiment^[^ ^13^ ^]^ under inhibitory conditions for the staphylococcal enzyme.^[^ ^11^ ^]^ As depicted in **Figure** **7**A, for growth control, planktonic *S. aureus* were detected (Figure 7B), and the typical clustered cocci morphology from biofilm of this bacterium was observed on the implant surface^[^ ^13^ ^]^ (Figure 7C). For M23‐PP/HAMS treatment (Figure 7D), the absence of bacterial growth (Figure 7E) and no signs of viable biofilm (Figure 7F)^[^ ^13^ ^]^ confirmed a bactericidal and antibiofilm mode of action of M23‐PP/HAMS NPs. In contrast, as depicted in Figure 7G, when the inhibitor of Hysa^[^ ^11^ ^]^ was added to the system, a bacteriostatic effect^[^ ^27^ ^]^ was attained with the corresponding reduction of 98% of the bacterial population (Figure 7H), while preserving the antibiofilm mode of action (Figure 7I). Altogether, these results confirm a bactericidal mode of action of M23‐PP/HAMS triggered by Hysa and support the idea of HAMS excipient acting as an antibiofilm agent discussed in Figure S8 (Supporting Information). Given these promising results, the efficacy of the HA‐based coated NPs in co‐culture was studied and is presented in Section 2.6.

### 2.6 Cytotoxicity and the Race for Surface between Bacteria and MC3T3

The wound healing assay gives information about the migration and proliferation of cells. We used this method, combined with live/dead staining, as an in vitro approach to assess the cytotoxicity of formulations toward the MC3T3 cell line. An intense uniform green fluorescence, which is produced by intracellular esterases‐mediated conversion of calcein AM in fluorescent calcein by metabolically active cells, suggested no toxicity of M23‐PP / HAMS and M23‐PP / HA toward MC3T3 cells (Figure 7). This result was further validated in the context of bone development as described in Figure S10 (Supporting Information), supporting the applicability of the formulations in bone tissue engineering.

**Figure 8:**
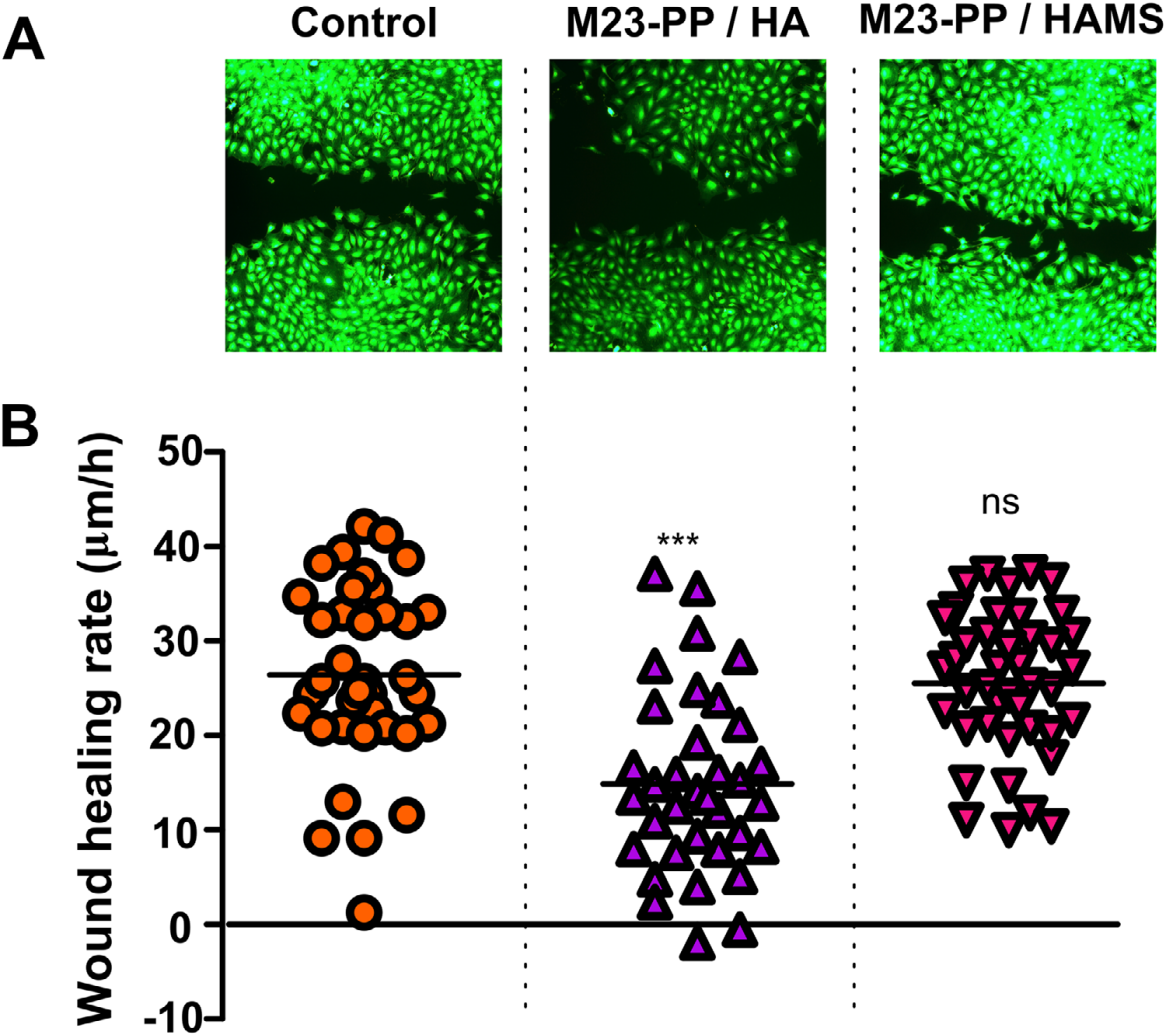
A) Live staining to confirm the absence of cytotoxicity by PPs, and B) rate of wound healing of MC3T3 in contact with a buffer control, M23‐PP/HA, M23‐PP/HAMS in the absence of bacteria. Values were obtained from 3 independent replicates. ^***^ *p* < 0.001, ns non‐significant (*p* ≥ 0.05).

The molecular size‐dependent effects of HA on cellular signaling, cell functional properties, cell morphology, and receptor binding are cell and context‐dependent.^[^ ^6^ ^]^ Here, the use of M23‐PP / HA NPs (containing LMW HA) decreased the rate of wound healing compared to the control (*p* < 0.001) (**Figure** **8**). However, unlike M23‐PP / HA, M23‐PP / HAMS treatment produced no changes in MC3T3 cell migration and proliferation (*p* ≥ 0.05) (Figure 8).

**Figure 9:**
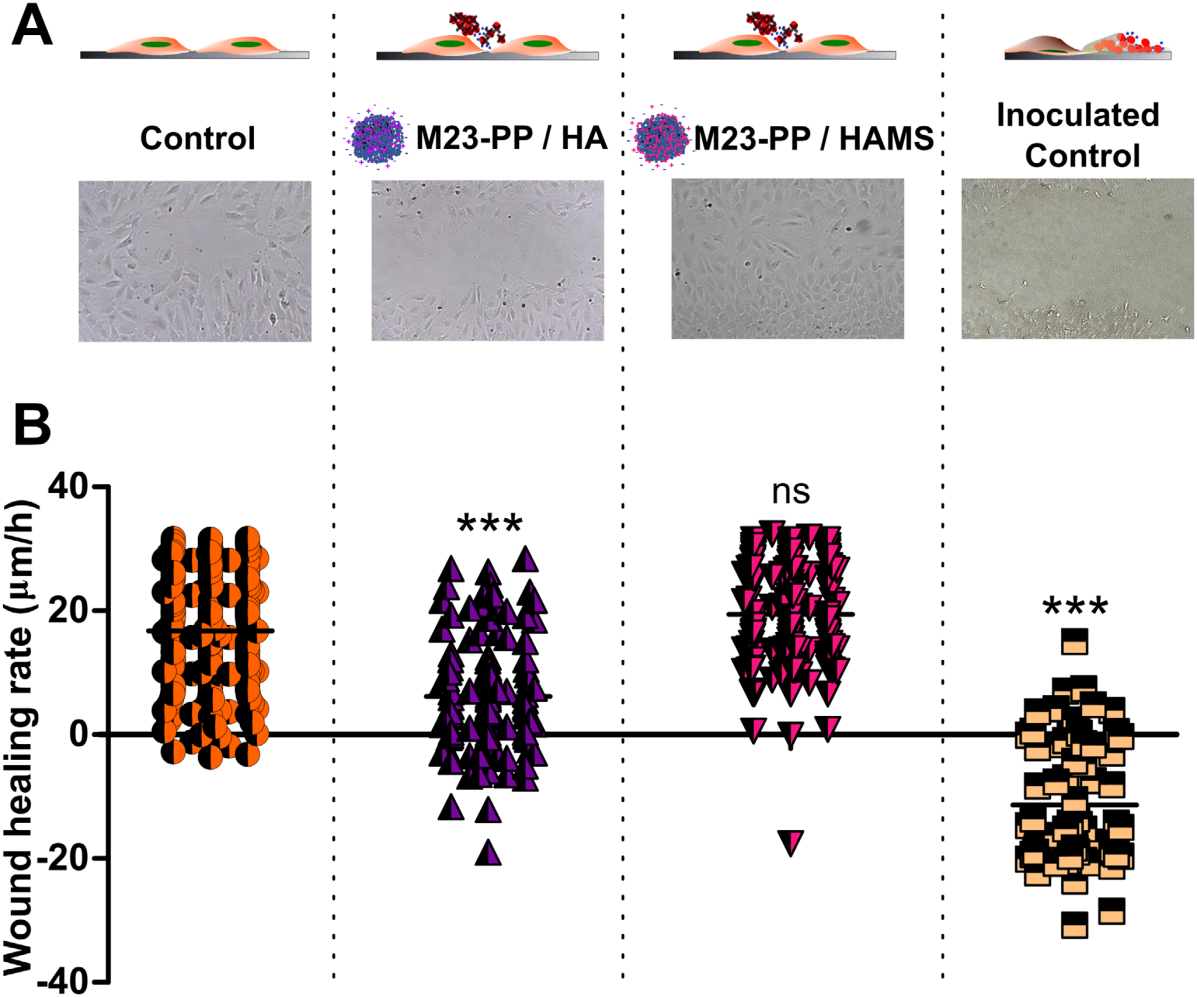
A) Depicts the micrographs of wound healing assay in a co‐culture of *S. aureus* ATCC 25923 and MC3T3 cells under the indicated treatments. B) Rates of healing (RoH) reached under the different treatments. Values were obtained from 3 independent replicates. ^***^ *p* < 0.001, ns non‐significant (*p* ≥ 0.05).

Surface colonization after implantation can be conceptualized as a contest between bacteria coming from the skin flora and tissue cells.^[^ ^28^ ^]^ To avoid bacteria winning the race for the surface, we propose the use of M23‐PP / HAMS NPs for the local targeted delivery of M23, potentially allowing for dosage reduction, minimizing side effects, while increasing effectiveness.^[^ ^29^ ^]^ Therefore, we tested the efficacy of our NPs system in a co‐culture experiment of MC3T3 and *S. aureus* ATCC 25923 when the NPs are present in a wound healing setup (**Figure** **9**). Bacteria grew to a final count of 8.89 ± 0.41 CFU mL^−1^, while MC3T3 were not able to proliferate, causing a wound retraction (*p* < 0.001) when both cells were growing together without any antimicrobial treatment (Figure 9). This highlights the ability of *S. aureus* ATCC 25923 to overgrow and colonize the surface, compared to MC3T3 cells. In contrast, after treatment of the co‐culture with either M23‐PP / HA or M23‐PP/ HAMS, conditions in which bacteria are at a big disadvantage compared to MC3T3, mammalian cells overgrew bacteria, causing wound healing (Figure 9), and complete bacterial kill (CFU mL^−1^ below the detection limit) was observed. In line with the cytotoxicity results discussed above, M23‐PP / HAMS treatment resulted in an RoH comparable to that of the control (*p* ≥ 0.05), while M23‐PP / HA showed a delay in the healing (Figure 9). These results prove the hypothesis that bacteria and mammalian cells compete on a race for the surface and validate our M23‐PP / HAMS formulation for its potential use to avoid bacterial growth under these conditions.

**Figure 10:**
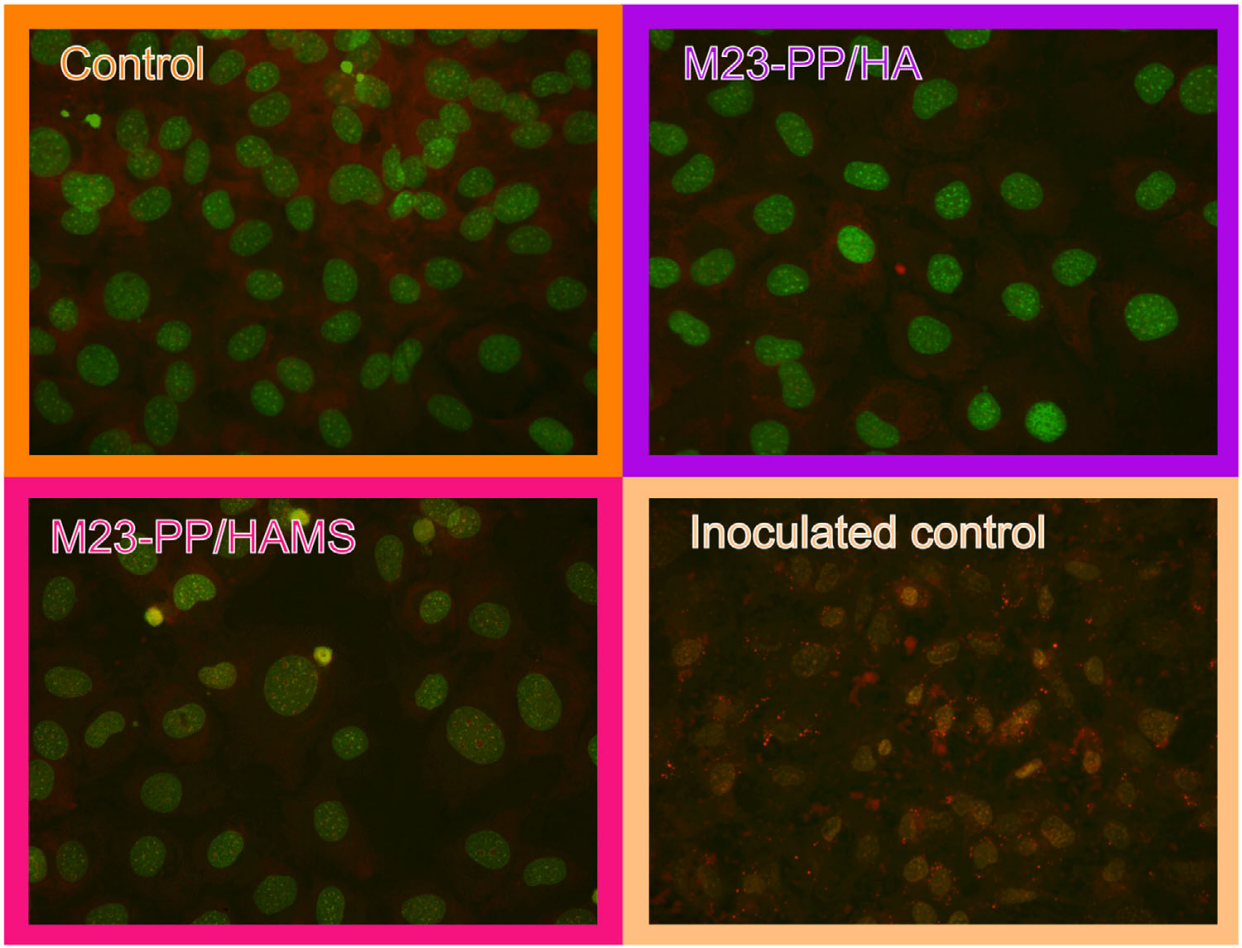
Acridine orange (AO) stained co‐culture of *S. aureus* and MC3T3 after treatment with the indicated samples.

Additionally, we applied our formulations in a confluent MC3T3 culture seeded with bacteria, for their potential use to avoid bacterial infection. As shown in **Figure** **10**, the MC3T3 cell line showed indications of unhealthy growth when bacteria were present. Namely, the morphology of both the nuclei and cytoplasm was distorted, while bacteria reached a final count of 8.85 ± 0.23 CFU/ ml (Figure 10, inoculated control). With the antimicrobial treatments, MC3T3 cells maintained their confluency, showing typical preosteoblast morphology (Figure 10, M23‐PP / HA and M23‐PP / HAMS), with no bacterial counts detected. These results highlight the potential of our formulations to avoid the infection of MC3T3 cells with *S. aureus* ATCC 25923.

## 3 Conclusion

Thiolated hyaluronic acid was used to develop redox‐responsive delivery systems, finding applications in biomedicine.^[^ ^30^, ^31^ ^]^ However, to the best of our knowledge, this is the first reported instance of its use in an enzyme‐responsive targeted delivery system. Within this gap in knowledge, we identified an opportunity to develop a material capable of extending storage time and preventing off‐target effects in our previously developed M23‐PP NPs.^[^ ^13^ ^]^ We hypothesized that using HAMS as a capping agent would render our system with increased stability and specificity toward *S. aureus* ATCC 25923.

The HAMS synthetized and characterized in our work proved to be a specific substrate for *S. aureus* Hysa, while avoiding degradation by Hyal from MC3T3 cells.

Polymer degradation and response to the milieu play a key role in the development of nanomaterials for controlled delivery.^[^ ^32^, ^33^ ^]^ The state‐of‐the‐art M23‐PP / HAMS obtained by ionotropic gelation provided an additional stimuli‐responsive barrier to the AP‐triggered switch, as demonstrated by in vitro release experiments and the mode of action of these NPs. Stability increased from 2.5 h at 4 °C for M23‐PP NPs^[^ ^13^ ^]^ to up to 7 days at 37 °C for M23‐PP / HAMS. Safety in the context of bone development and efficacy on ´the race for the surface´ between MC3T3 and *S. aureus* ATCC 25923 in co‐culture experiments showed promise for the application of HAMS / M23 PP NPs as antimicrobial agents to avoid infection by *S. aureus*.

Implant‐associated infections remain a devastating complication, carrying significant morbidity for patients and a substantial economic impact on healthcare systems. To counter this threat, several evolving approaches are applied to prevent infections from happening.^[^ ^34^ ^]^ However, many of these approaches carry the risk of promoting antibiotic resistance as a result of antibiotic overuse. We propose that HAMS / M23 PP NPs technology can be used as a reinforcement of the current practices, offering an added layer of protection against the ongoing challenge of implant‐associated infections.

Our new enzyme‐responsive excipient switchable by *S. aureus* provides a gating strategy to prevent implant‐related infection. This is key to avoiding antibiotic overuse and, therefore, minimizing the risk of resistance development.

## 4 Experimental Section

### Materials

Sodium borohydride (NaBH_4_), sodium chloride pellets; cetyltrimethylammonium bromide (CTAB); sodium hydroxide; sodium polyphosphate (PP, Graham's salt, n = 25); violet crystal ≥90% purity anhydrous basis; alkaline phosphatase (AP) from bovine intestinal mucosa (≥10 U mg^−1^ solid); malachite green oxalate (MLG); ammonium molybdate tetrahydrate; fetal bovine serum (FBS); magnesium chloride (MgCl_2_); D‐glucose; 4‐nitrophenyl phosphate and p‐nitrophenol were purchased from Sigma‐Aldrich, Austria. 5,5‐Dithio‐bis‐(2‐nitrobenzoic acid); L‐cysteine; hyaluronate lyase from *Streptomyces hyalurolyticus* (S‐Hysa); type I‐S hyaluronidase from bovine testes (BT‐Hyal); N,N‐dimethylacetamide (99.8%), ColiRollers plating beads, and 2‐amino‐2‐methyl‐1‐propanol were purchased from Merck, Germany. DL‐Dithiothreitol; LIVE/DEAD viability/cytotoxicity assay kit (Green/Deep Red); MEM α, nucleosides, no ascorbic acid; MEM α (nucleosides, no phenol red), and acridine orange (AO) were provided by Thermo Fisher Scientific, Austria. DL‐Mercapto succinic acid (99%) and acetyl chloride (98%) were purchased from ACROS Organics. N,N‐Dimethylformamide DMF (≥99,5%); Spectra/por 6 dialysis membrane, MWCO 5 kDa; Spectra Por Float‐A‐Lyzer dialysis tubes cut‐off: 0.1–0.5 KDa; 4‐(2‐hydroxyethyl)‐1‐azineethanesulfonic acid (HEPES); glycerol (99.5%); agar, and tryptic soy broth (TSB) were purchased from Carl Roth, Germany. Diethyl ether (technical) was provided by VWR, International. Culture‐Insert 2 well by Ibidi, Germany; low molecular weight hyaluronic acid (HA) sodium salt (Cat No. FH76335, Poly(β‐glucuronic acid‐[1→3]‐β‐N‐acetylglucosamine‐[1→4]); CAS 9067‐32‐7, min. 95.0%, LMW 10000–50000 Da, $\bar{M}$ *_w_* = 21400 Da determined via static light scattering, was produced by Biosynth, Austria. *E. coli* BL21 Gold (DE3) (Stratagene, La Jolla, CA, United States). MycoStrip – Mycoplasma Detection Kit (Ivivogen, AT). All other reagents were of analytical grade and obtained from commercial sources.

### Cell Lines, Growth Conditions, and Enzybiotic

*Staphylococcus aureus* ATCC 25923 was provided by the Biofilm Lab (Experimental Orthopaedics, University Hospital for Orthopedics and Traumatology, Medical University Innsbruck). Growing conditions involved TSB at 37 °C under shaking. Glycerol stocks of bacteria were stored at −20 °C.

Preosteoblast‐like cell line MC3T3‐E1 Subclone 4 (ATCC CRL‐2593, RRID: CVCL_5440) was purchased from the ATCC collection. The cell line was mycoplasma‐free as tested by MycoStrip – Mycoplasma Detection Kit (Ivivogen, AT). Growing conditions were incubation with MEM α, nucleosides, no ascorbic acid, supplemented with FBS (Supplemented MEM) at 37 °C in 5% CO_2_ atmosphere and fed every 2 to 3 days up to 90–100% confluency. DMSO stocks were maintained in liquid nitrogen.

Recombinant M23LST(L)_SH3b2638A (M23) (27.414 KDa, M23) composed of an M23 endopeptidase domain from lysostaphin and an SH3b cell wall binding domain (CBD) from phage 2638A endolysin was expressed in *E. coli* BL21 Gold (DE3) and purified as previously reported.^[^ ^35^ ^]^

### Synthesis of Hyaluronic Acid Mercaptosuccinate

Hyaluronic acid was esterified with DL‐mercaptosuccinic acid using a two‐step reaction, modifying the method previously published.^[^ ^16^, ^17^ ^]^ First, S‐acetyl mercaptosuccinic anhydride was obtained by mixing 60.4 g of mercaptosuccinic acid (0.4 mol) with 150 mL of acetyl chloride (2.0 mol) and heating to reflux for 3h. The obtained product was concentrated using a Rotary evaporator (Heildolph 2, G3), and precipitated into cold diethyl ether. The formed solid was filtered, washed with diethyl ether, and dried overnight under reduced pressure at room temperature. For the esterification step,^[^ ^16^, ^17^ ^]^ 1 g of hyaluronic acid was mixed and dehydrated with 13.33 g of S‐acetyl mercaptosuccinic anhydride (0.08 mol) at 75 °C under reduced pressure for 2.5 h. Afterward, 30 mL of dimethyl acetamide was added, incubated under stirring at 50 °C for 2 h, with further incubation under reflux for 48 h at 100 °C, as shown in Scheme 1. The dark brown component obtained was precipitated with precooled diethyl ether in a ratio of 1:10 by agitation (30 min), filtered, washed with cold diethyl ether, and dried under vacuum. In a further step (Step 2 in Scheme 1), deprotection of the thiol group was achieved in basic conditions. The optimized acetyl removal reaction is described in the supporting information (Figure S1, Supporting Information). The suspension obtained was purified by dialysis (Spectra/por 6 dialysis membrane, MWCO 5 kDa) and freeze‐dried for further analyses.

To reduce disulfide bonds, the obtained product was dissolved in DMF (30mL), mixed with 10 mg of DL‐dithiotheritol, and incubated under N_2_ atmosphere overnight. The brown solid obtained after precipitation with diethyl ether was dried under vacuum at 40 °C.

### Characterization of Hyaluronic Acid Mercaptosuccinate

To confirm the structure of the produced ester, ^1^H‐NMR measurements were executed on a “Mars” 400 MHz Avance 4 Neo spectrometer from Bruker Corporation (Billerica, MA, 400 MHz). The spectra were recorded in deuterium oxide (D_2_O) solution. For the measurements, ≈4 mg of the freeze‐dried HAMS were dissolved in 0.65 mL of D_2_O. The chemical shifts were reported in parts per million, and the center of D_2_O served as the internal standard (δ 4.79 ppm).

Free thiol (free _SH_) and total thiol (total _SH_) contents were determined by the Ellman's assay before and after reduction of thiols with NaBH_4_, respectively.^[^ ^36^ ^]^ Briefly, *ca* 0.5 mg mL^−1^ of HAMS dissolved in Ellman´s buffer were mixed with 5,5‐dithio‐bis‐(2‐nitrobenzoic acid). After incubation at 37 °C for 90 min, absorbance was recorded at 450 nm using a Tecan Spark microplate reader (M‐200 spectrometer, Tecan, Grödig, Austria). To calculate µmol of thiol per gram of sample, a calibration curve was built using L‐cysteine as a standard. Disulfide content was obtained by subtracting free _SH_ from total _SH_. These measurements were performed in triplicate.

Solubility was determined according to the OECD standard with some modifications.^[^ ^17^ ^]^ A stepwise procedure was performed to determine the range of solubility of the samples. For this, 10 mg of pulverized samples were incubated (10 min, 25 °C, 1000 rpm) with increasing amounts of water (from 100 to 1 mL). Solubility was checked visually after each water addition/incubation cycle.

The molar masses of the precursor HA and the HAMS were determined using static light scattering (SLS) with a Zetasizer Nano ZSP (Malvern Instruments, Worcestershire, UK). The measurements were conducted at a laser wavelength of 633 nm and a temperature of 25 °C, with a refractive index increment (dn/dc) of 0.165 mL g^−1[^ ^37^ ^]^ for light scattering analysis. The polymers were dissolved in demineralized water at concentrations ranging from 2.00 to 0.0625 g L^−1^.

pKas of HAMS were determined by potentiometric titration as described in supporting information (Figure S2, Supporting Information).

### Hyaluronidase, Hyaluronate Lyase, and Enzymatic Cleavage of HA and HAMS

Hyaluronidase activity produced by MC3T3 and *S. aureus* ATCC 25923 was determined with a turbidimetric method as previously reported.^[^ ^38^ ^]^ For this, the bacteria or mammalian cells were grown under optimum conditions and centrifuged. The spent media were supplemented with 0.125 mg mL^−1^ of either HA or HAMS in 0.2 m acetate buffer (pH 6) containing sodium chloride 0.15 m and incubated under shaking at 37 °C and 300 rpm. The reaction was stopped with CTAB 2.5% dissolved in 2% sodium hydroxide. Turbidity was determined at 600 nm using a plate reader (Tecan Spark, Tecan Trading AG, Switzerland). A calibration curve was built using different concentrations of BT‐Hyal and 0.125 mg mL^−1^ HA substrate.

Kinetic of HA and HAMS degradation was studied as reported elsewhere.^[^ ^39^ ^]^ BT‐Hyal and *Streptomyces hyalurolyticus* (S‐Hysa), respectively, were used as a surrogate of mammalian and bacterial enzymes. Degradation of different concentrations of the substrates over time was studied, and V_0_ was determined for every concentration using 500 UI mL^−1^ of either BT‐Hyal or S‐Hysa surrogates. Modeling and prediction of kinetic parameters were performed with GraphPad Prism 5 (GraphPad Software, Inc., San Diego, CA, USA). Kinetics of HAMS degradation by MC3T3 and *S. aureus* ATCC 25923 spent was also characterized.

The characterization of HAMS as an inhibitory ligand for BT‐Hyal was carried out as described in Figures S3–S5 (Supporting Information)

### Coating of M23‐PP NPs with HAMS or HA, and Particle Characterization

M23‐PP NPs obtained as previously described^[^ ^13^ ^]^ were dropped under stirring (1400 rpm, 24 °C) in HAMS or HA dissolved in water at pH 2, in ratios ranging from 1:2 to 1:20 of PP to HA‐based polymers. Zetasizer Nano (ZSP, Malvern Instruments, Worcestershire, UK) was used to measure particle size distribution, mean dynamic particle size (DLS), and zeta potential of each sample at 24 °C. Zetasizer Software 8.02 Copyright 2002–2021 Malvern Panalytical provided the required data fitting algorithms and analysis.^[^ ^40^ ^]^

Visual confirmation of nanoparticles’ presence was conducted by energy‐filtered transmission electron microscopy (EFTEM). 200 mesh formvar‐coated copper grids (Plano, Wetzlar, Germany) were used for mounting 5 µL of each freshly prepared sample. For imaging the particles, a Zeiss Libra 120 microscope (Carl Zeiss AG, Oberkochen, Germany) equipped with a 2 × 2 k high‐speed camera (Troendle, Germany) and the ImageSP software (Troendle, Germany) were used.

The HAMS and HA‐coated M23‐PP NPs (M23‐PP/HAMS NPs and M23‐PP/HA NPs, respectively) were characterized for size distribution and zeta potential after preparation and over incubation for 1, 3, and 7 days at 37 °C under shaking (150 rpm). These experiments were performed in at least 6 replicates.

Minimum inhibitory, bactericidal concentration, and antibiofilm forming concentrations (MIC, MBC, and MAC, respectively) were determined by the broth dilution method.^[^ ^41^, ^42^ ^]^ Briefly, serial two‐fold dilutions of M23‐PP/HAMS and M23‐PP/HA NPs were added to a 96‐well plate and supplemented with 10^5^ CFU mL^−1^ of *S. aureus* ATCC 25923 in TSB+1% glucose. After 16 h at 37 °C, MIC was defined as the concentration showing no turbidity at 620 nm. From wells showing no turbidity, 100 µL of the sample was plated on TSA and incubated for 48 h at 37 °C. MBC was defined as the dilution showing no growth after plating, which corresponds to 99.9% of killing. Finally, MAC was determined by biofilm staining using violet crystal (100 µL, 0.1% m V^−1^) after removal of planktonic bacteria.

Phosphate release triggered by AP and BT‐Hyal was studied as previously reported.^[^ ^43^ ^]^ In a dialysis tube of 0.1–0.5 kDa cutoff (donor chamber), 1 mL of NP formulations was added and placed in a Falcon tube (acceptor chamber) containing 10 mL of HBS buffer (control) and AP 0.006 DEA mL^−1^ in HBS or BT‐Hyal 10 UI mL^−1^. Two independent replicates of this setup were incubated at 37 °C. Samples (100 µL) were taken from the acceptor chamber after 1, 2, 24, 96, and 144 h. To determine phosphate release, MLG reagent was mixed with samples in equal parts, and absorbance at 630 nm was determined with a Tecan instrument.^[^ ^43^ ^]^ A calibration curve was made with KH_2_PO_4_ to calculate the free phosphate released. The size distribution of samples residing in the dialysis bags was determined by dynamic light scattering at the end of the experiment.

### Hysa‐Triggered Efficacy of M23‐PP/HAMS

A total of 10^5^ CFU mL^−1^ of *S. aureus* in TSB+1% glucose was grown on stainless‐steel discs (growth control), in the presence of M23‐PP/HAMS NPs (M23‐PP/HAMS treatment), and using serum as an inhibitor of Hysa^[^ ^11^ ^]^ (M23‐PP/HAMS treatment in the presence of Hysa inhibitor). After overnight incubation at 37 °C under shaking (100 rpm), plating of planktonic bacteria was performed, while the implants were fixed using 0.5 mL of 2.5% glutaraldehyde for 4 h at room temperature. Following fixation, dehydration was carried out utilizing a graded ethanol series (50%, 70%, 80%, and 99.9%), with each step lasting 5 min. The dehydrated samples were then air‐dried and mounted on aluminum pins by means of a carbon adhesive. The samples were subsequently sputter‐coated with gold for 45 s using an automatic sputter coater (Agar Scientific Ltd., Stansted, UK) and imaged through a scanning electron microscope (SEM, JSM‐6010LV, JEOL GmbH, Freising, Germany).^[^ ^13^ ^]^

### Wound Healing and Cytotoxicity

The impact of M23‐PP/HAMS and M23‐PP/HA on MC3T3 cell migration and proliferation was studied with the wound healing assay.^[^ ^44^ ^]^ Ibidi culture‐Inserts 2 wells were attached to 24 well plates and seeded with 70 µL of a suspension containing 2.5 105 MC3T3 cells mL^−1^. After growth to confluency for 24 h at 37 °C in 5% CO2 atmosphere, the inserts were removed and samples (156 nm in M23 concentration) were applied in PBS for 2 h. After this, the cells were washed with PBS and further incubated for 24 h. The negative control consisted of cells treated with PBS only. Images of the wound were taken with Leica Fluorescence Leica DM IL LED Fluo 11521265 Microscope (LAS X 3.7.6 software) before sample application (t0) and after 24 h of incubation (t24). Between 10 and 15 measurements of each gap were taken using ImageJ.^[^ ^45^ ^]^ The rate of healing (RoH, µm h^−1^) was calculated with the following formulation of Eq. (1)

(1) RoH=(Wf−Wi)/t

Where Wf is the wound gap at t24, Wi is the wound gap at t0, and t is the total time of incubation between the measurement of the two gaps (26 h).

LIVE/DEAD Viability/Cytotoxicity Assay Kit (Green/Deep Red) staining was performed after the experiments to better visualize the gap and assess cytotoxicity of the formulations. MC3T3 cells were incubated for 30 min with MEM α (nucleosides, no phenol red) supplemented with calcein AM (Ex/Em ≈495/≈515 nm) and SYTOX Deep Red Nucleic Acid Stain (Ex/Emmax 660/682 nm) at a final concentration of 2 and 4 µm, respectively. With this staining method, live cells emit a green fluorescence from calcein dye, while dead cells emit a red fluorescence from SYTOX Deep Red staining. Cells were imaged after washing with MEM α (nucleosides, no phenol red). Experiments were performed on at least 3 independent replicates.

Biocompatibility of the formulations in the context of bone development was carried out as described in Figure S10 (Supporting Information)

### Co‐Culture Experiments

Two different co‐culture setups were run. i) Cell migration and proliferation of MC3T3 in the presence of *S. aureus* ATCC 25923. For this, the wound healing experiment was performed as described above by adding 10^5^ CFU mL^−1^ of *S. aureus* ATCC 25923, mimicking the flora of clean skin.^[^ ^46^ ^]^ The growth medium used was supplemented MEM α, and the inoculation with bacteria was performed immediately after the removal of the wound insert. M23‐PP/HA and M23‐PP/HAMS NPs were separately applied to the co‐cultures and incubated for 2 h, followed by antimicrobial formulation removal and further incubation for 24 h at 37 °C in 5% CO_2_ atmosphere. For the calculation of RoH (µm h^−1^), images were taken after sample removal and 24 h later. Negative control consisted of MC3T3 grown in supplemented MEM α, while the inoculated control consisted of MC3T3 co‐cultured with *S. aureus* ATCC 25923 under the same conditions, but no antimicrobial treatment. ii) Additionally, a confluent culture of MC3T3 was seeded with *S. aureus* ATCC 25923 and treated for 2 h separately with M23‐PP HA and M23‐PP‐HAMS NPs. After treatment, cells were fixed with paraformaldehyde 2% in PBS for 20 min, washed with PBS once, and incubated for 30 min with DAPI (10 µg mL^−1^). After three washing steps, AO (1 mg mL^−1^) in PBS was incubated with the cells for 20 min, followed by two washing steps with water.^[^ ^29^, ^47^ ^]^ Cells were imaged as described in the previous section, using a DAPI filter. Under these conditions, bacterial cells exhibited a bright orange/red color, eukaryotic cells showed a less bright red in the cytoplasm and a distinct green nucleus. For all sample/treatment combinations, serial dilutions from separate wells were plated on TSA, incubated at 37 °C for 48 h, and subsequently counted to determine the colony‐forming units (CFU mL^−1^) of *S. aureus* ATCC 25923.^[^ ^43^ ^]^ Experiments were run in 3 independent replicates, with counts performed in additional technical duplicates.

### Statistical Analysis

GraphPad Prism 5 (GraphPad Software, Inc., San Diego, CA, USA) was used for data analysis. To assess the normality of data distribution, D'Agostino‐Pearson, Shapiro‐Wilk, and Kolmogorov‐Smirnov (with Dallal‐Wilkinson‐Lilliefor P value) tests were run, concluding by looking at all the results. For Gaussian‐distributed data, one‐way ANOVA and two‐way Bonferroni tests were used, while for Beta‐distributed data, the Kruskal‐Wallis test with Dunn's post hoc was applied. Statistical significance was set at *p* < 0.05.

## Conflict of Interest

The authors declare no conflict of interest.

## Author Contributions

M.B.M. performed funding acquisition, conceptualization, experimental design, overall data analysis, and results interpretation, writing the original draft, corrections, and proofreading. S.M. performed chimeric phage endolysins production, manuscript correction, and proofreading. A.K. supported work with bacteria and proofreading. D.G. supported in phosphate release experiments and proofreading. A.S. performed EFTEM analyses and proofreading. D.C.C‐H. performed proofreading and provided access to laboratory facilities. G.K. performed NMR analysis, supported in synthesis, manuscript correction, and proofreading. I.P. supported in mammalian cell work, HPLC, manuscript correction, and proofreading. M.J.L. performed chimeric phage endolysins conceptualization, funding acquisition, manuscript correction, and proofreading. M.S. performed chimeric phage endolysins conceptualization and supervision, funding acquisition, manuscript correction, and proofreading. S.Z. performed writing – review and editing, funding acquisition, and supervision. A.B.‐S. performed writing – review and editing, funding acquisition, and supervision.

## Supporting information



Supporting Information

Supporting Information

## Data Availability

The data that support the findings of this study are available in the supplementary material of this article.
