# Supplementary material for: Thiolated Hyaluronic Acid: A Gateway for Targeted Killing of Staphylococcus aureus on the Race for Surface Colonization
Source: Adv Healthc Mater. 2025 Sep 12;15(2):e02890. doi: 10.1002/adhm.202502890 (PMC12805623; doi:10.1002/adhm.202502890)

**Electronic Supplementary Information**

**Figure S1.** Deprotection of S-acetyl mercaptosuccininate hyaluronic acid was optimized by treating 1 mg/ml of thiolated HA with NaOH (3.16 mM) pH 11. 5 for different times. To quantify the released acids after treatment, 5 ml of thiolated HA treated at different conditions were added into a dialysis tube (Dialysis systems Spectra/Por ® Float-A-Lyzer ® G2 filling volume 5 ml, 100–500 Dalton). A Falcon tube containing 50 ml of water was used as a receptor chamber. Dialysis was performed under shaking for 3 days at 100 rpm and at room temperature. The contents of the receptor chamber were lyophilized, and water resuspended for HPLC analyses according to Reuter et al, 2015. ^[1]^ Standard solutions of acetic acid, S-acetyl-mercapto-succinic acid and 2-mercapto-succinic acid were prepared. The system consisted of a Hitachi Chromaster (Tokyo, Japan) equipped with a 5160 pump, 5260 autosampler, 5310 column oven, and 5430 photodiode array UV detector. In brief, the stationary phase was a NUCLEOSIL 100-5 C18 column (100 × 4.6 mm, 5 μm), the column oven was set to 30 °C and the injection volume was 20 µL. Gradient elution was performed as follows: flow rate of 1.5 mL/min; 0-10 min; linear gradient from 80% A/20% B to 40% A/60% B; flow rate of 1.5 mL/min; 10-12.5 min; isocratic elution with 40% A/60% B; flow rate of 1.5 mL/min ; 12.5-12.6 min; linear gradient from 40% A/60% B to 80% A/40% B (eluent A: 10 mM KH2PO4 pH 2.4; eluent B: acetonitrile). The detection wavelength was set to 210 nm. A plateau of release acetic acid was reached after 45 min of treatment, with no peaks observed for 2-mercapto-succinic acid. This indicates that the treatment was sufficient for deprotection without causing hydrolysis of the thiolated component.

**
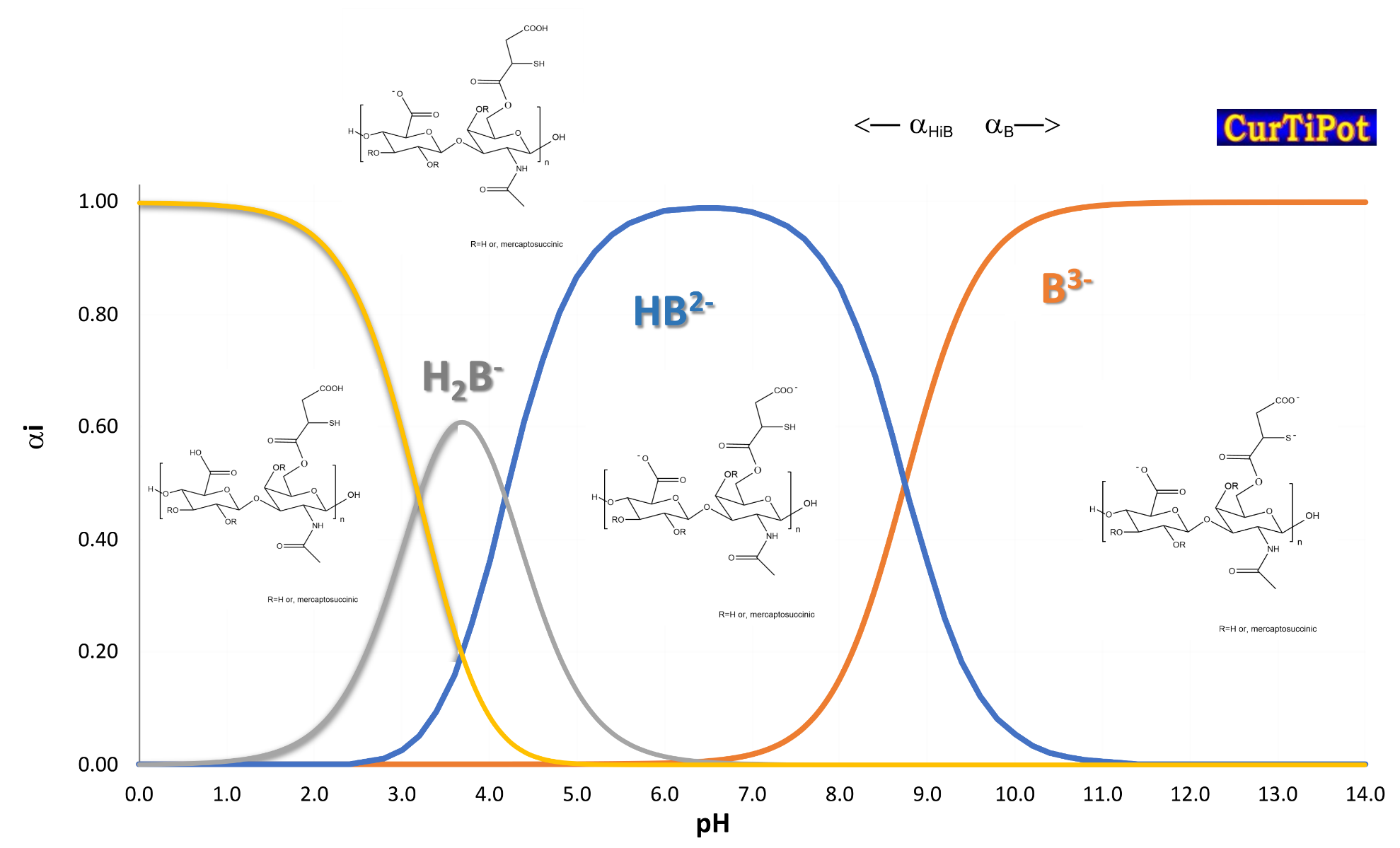
**

**Figure S2.** Distribution diagrams of protonated and deprotonated species in HAMS. Potentiometric titration of 1 mg/ml mercaptosuccinate modified HA (HAMS) dissolved in 10 ml HCl 0.001 M and NaCl 0.1M (constant ionic strength) was performed in triplicate. Prediction and simulations for designing titration experiments, as well as pKa estimations were made by using CurTiPot program (Version 4.3.1 option i. May/2021. for Microsoft Excel^®^ , Copyright © 1992 – 2021, Prof. Ivano G.R. Gutz. [gutz@iq.usp.br](mailto:gutz@iq.usp.br)). All curves were computed at a fixed ionic strength of 0.1. The distribution shows the existence of H_3_B, H_2_B^-^, HB^2-^ and B^3-^ species.

Protonation constants (Table 1, main manuscript) could be approximated from the diagram of species, since at the upper intersections the points [HBn] equals to [HBn-1] and this occurs at a pH values equal to the protonation constants. ^[2]^ The correlation of species from the diagram were suggested considering HA ^[3]^ and mercaptosuccinic acid (MS) pKa constants. ^[4]^ The shift of pKa_3_ (Thiol-thiolate equilibrium) of MS from 10.64 ^[4]^ to 8.8 in HAMS may be explained by the withdrawing effect of carboxylate neighboring groups in HAMS molecule, ^[5]^ confirming the binding of MS to the HA backbone.

As will be further discussed, the high pKa_3_ in HAMS may have an impact in the ability of this formulation to inhibit Hyal from mammalians (**Figure S3).**

**
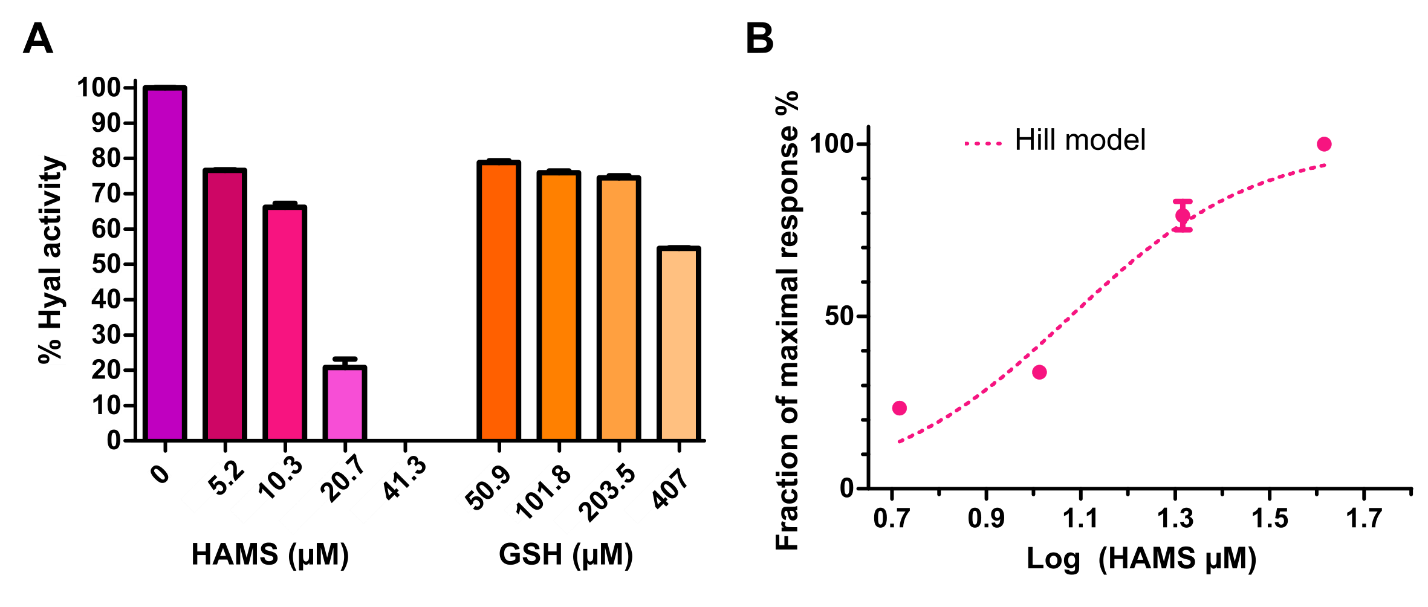
**

**Figure S3. (A)** To check the hypothesis that HAMS acts as inhibitor of mammalian Hyal, BT-Hyal (1 UI/ml) was preincubated (10 min at 37°C) with increasing concentrations of HAMS and GSH (used as inhibitory model). Thereafter, 0.125 mg/mL (36.6 µM) of hyaluronic acid in 0.1M acetate buffer pH 5.5 containing 0.15 M NaCl were incubated at 37 ◦C for 15 min and the reaction stopped by adding CTAB 2% in NaOH 2%. A dose-dependent inhibition of Hyal by HAMS and GSH was obtained. Given that the enzymatic conditions are performed at a pH below pKa_3_ from HAMS, it can be suggested that thiol groups from the backbone of HAMS will be more predominant than their conjugated base (**Figure S2**). Available thiol groups may interact through hydrogen bonding with the hydrophobic amino acid residues of Hyal that assist the ligand binding, therefore exerting inhibition. ^[6]^ A dose of 41.3 µM of HAMS was able to completely inhibit BT-Hyal (1 UI/ml), which is promising for developing a targeted release strategy as will be further explained in Section 2.3 of the main manuscript. (**B**) Log HAMS concentration vs the fraction of maximal response showed a good fit to the Hill model (R²=0.9467), with a HAMS IC_50_ of 11.99uM and a Hill coefficient of 2.02. According to the model assumptions, this corresponds to cooperative binding of the inhibitor to the enzyme. ^[7]^

**
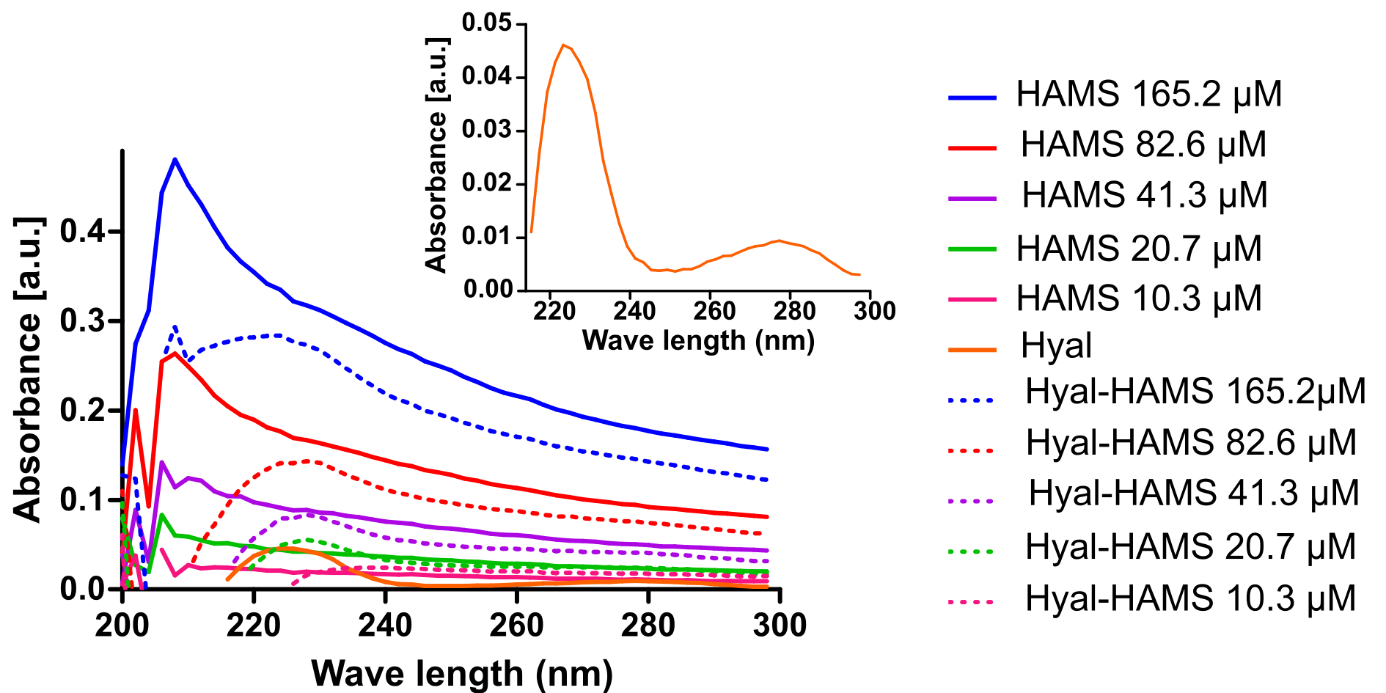
Figure S4.** To gain deeper insights into the interaction of HAMS and BT-Hyal, binding at saturation of the ligand was studied by varying the concentration of HAMS (162.2 µM to 10.3 µM) for 50 UI/ml of BT-Hyal and recording the UV absorbance for every enzyme/ligand combination. BT-Hyal (Inset in **Figure S4**) showed a strong peak at 224 nm associated with the peptide bond, and the aromatic peak at 278 nm, which correlates with Tyr and Trp content. ^[8]^ An increase in the peak at 224 nm was observed with incrementing concentrations of HAMS ligand, accompanied by a concomitant disappearance of the band at 278 nm. These results are in good agreement with a putative interaction between the thiolated ligand through hydrogen bonding with Tryptophan at the active site of BT-Hyal, as previously suggested. ^[6]^ As will be further discussed (**Figure S5**), this interaction seems to cause an irreversible inhibitory effect in BT-Hyal.

**
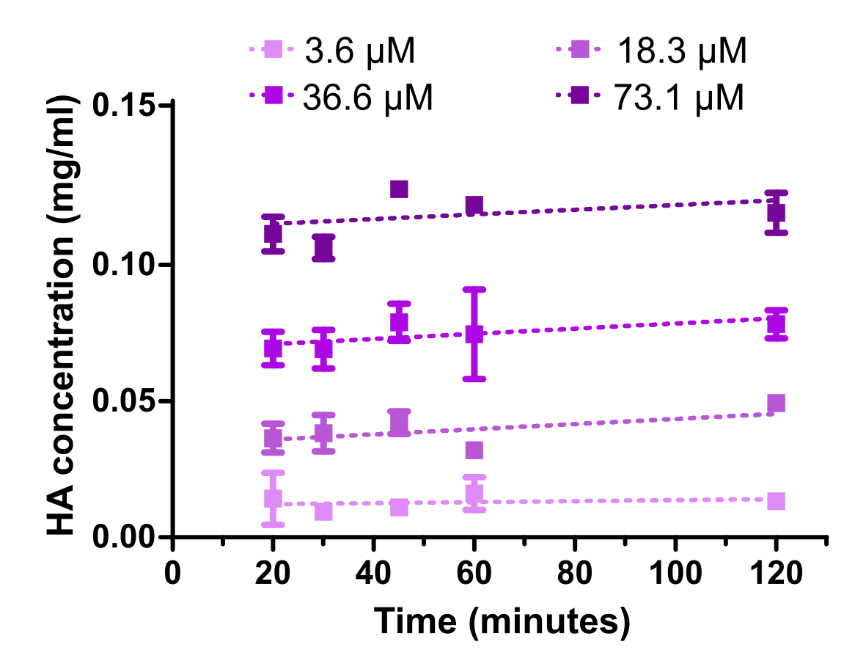
**

**Figure S5.** To further understand the implication of HAMS interaction with BT-Hyal, the enzyme (1 UI/ml) was preincubated (10 min at 37°C) with 20.7 µM (0.125 mg/ml) of HAMS. Afterwards, increasing concentrations of HA substrate ranging from 3.6 µM to 73.1 µM (0.025 to 0.5 mg/ml) were incubated in acetate buffer at 37 ◦C during different times, and the reaction stopped by adding CTAB 2% in NaOH 2%. The unchanged reaction velocity across increasing HA substrate concentrations points to HAMS functioning as an irreversible inhibitor of BT-Hyal. This result, paired with the high Hill constant found (**Figure S3**), suggests a cooperative binding of HAMS inducing changes that destabilize the active form of BT-Hyal. ^[7]^


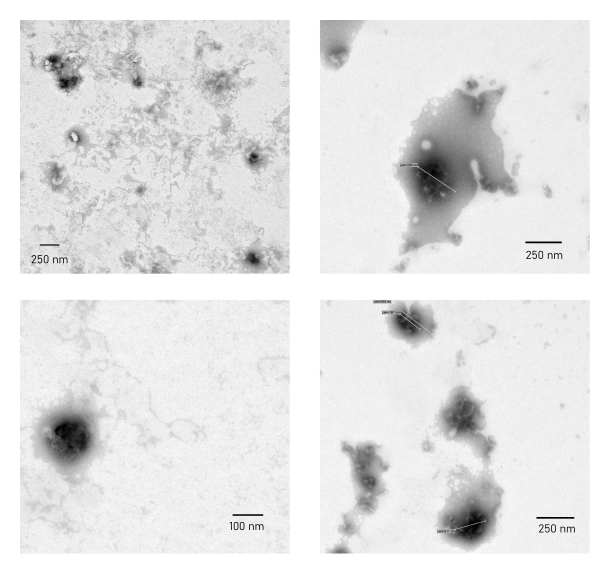


**Figure S6.** M23-PP / HAMS NPs characterized by TEM revealed irregular morphology and a broad size distribution. The mean particle values were in coincidence with those obtained by DLS (**Figure 5B**, main manuscript). However, the results should be interpreted with caution, as sample preparation seems to introduce artifacts that interfere with accurate measurements.


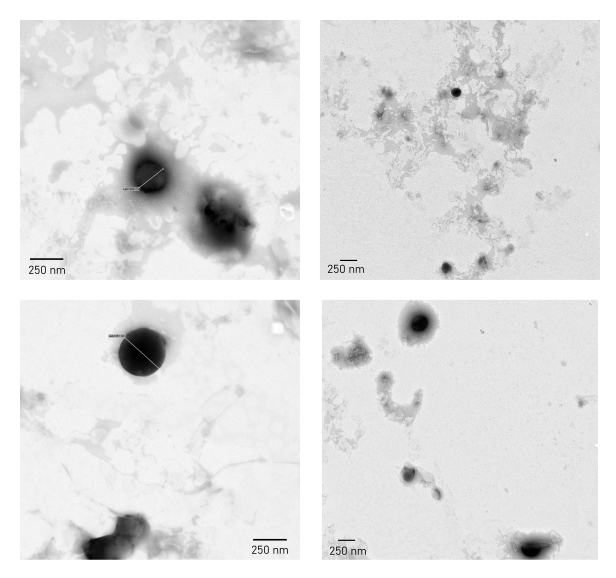


**Figure S7.** M23-PP / HA NPs characterized by TEM showed irregular morphology and a broad size distribution. The mean particle values were in coincidence with those obtained by DLS (**Figure 5B**). However, the results should be interpreted with caution, as sample preparation seems to introduce artifacts that interfere with accurate measurements.


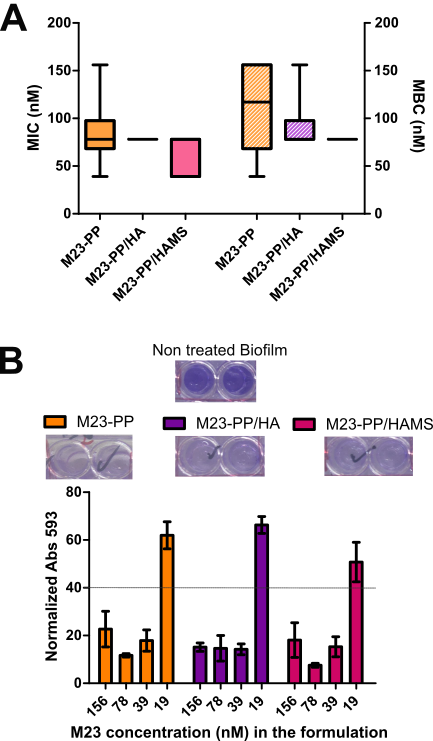


**Figure S8**. (A) There was no statistical significance between the MIC and MBC values among M23-PP, M23-PP/HA, and M23-PP/HAMS (p ≥ 0.05). (B) Strong prevention of biofilm formation was obtained after treatment with the NPs, as visualized by staining with violet crystal. After dye extraction and absorbance measurement at 593 nm, the minimum anti-biofilm concentration (MAC) was defined as the concentration that resulted in more than a 40% reduction in absorbance. ^[9]^ All the formulations showed a MAC of 39 nM in M23. Interestingly, the MAC values fell below the MIC and MBC for all the NPs formulations, suggesting antibiofilm activity at low concentration when the enzymes are not able to kill the bacteria. We attribute this result to the antibiofilm properties of the excipients rather than the effect of M23 phage endolysin. ^[10,11]^ Values presented are means of at least 6 replicates ± SD.

**
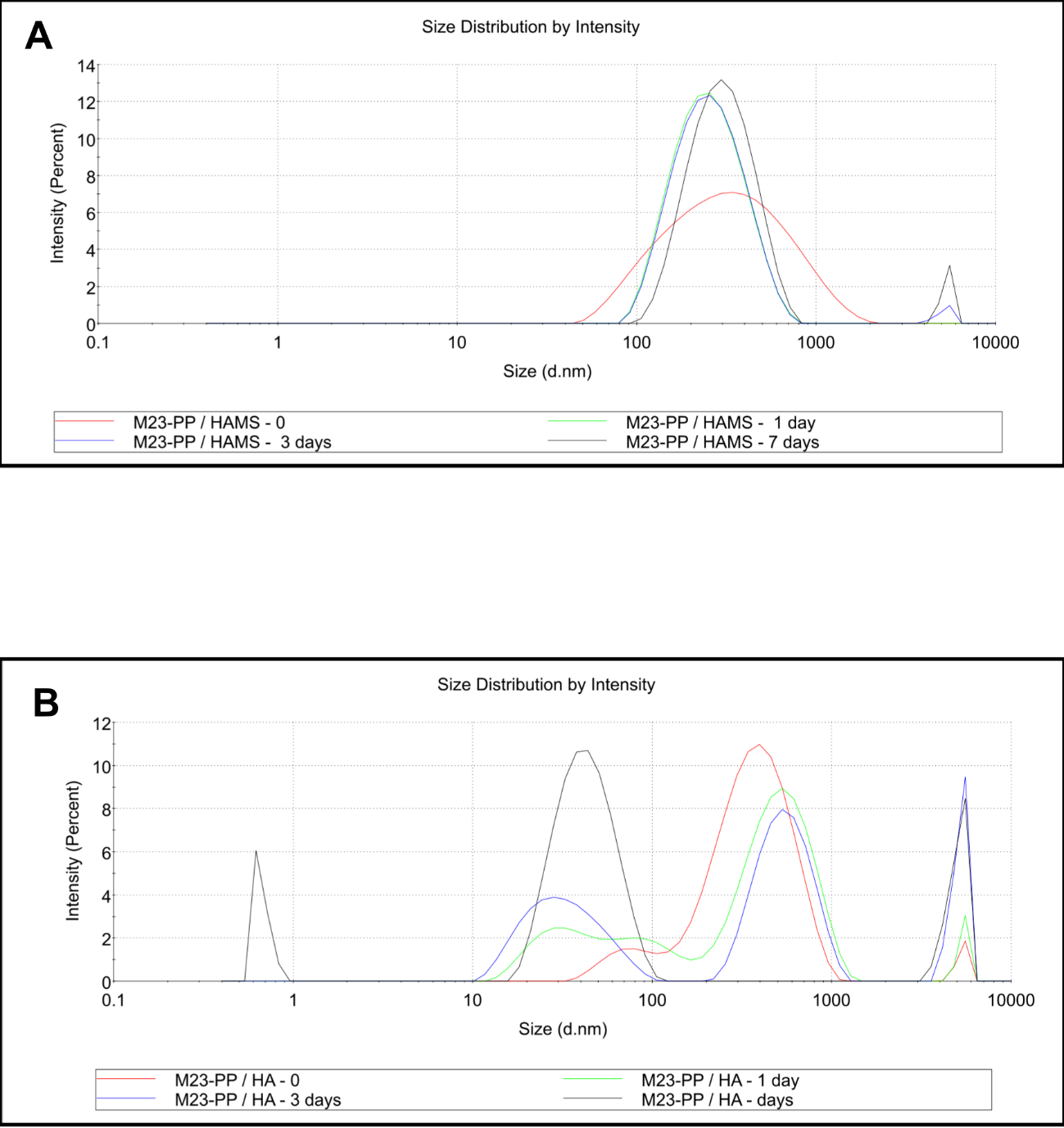
**

**Figure S9**. (A) M23-PP / HAMS NPs maintained their size distribution over storage for 7 days at 37°C, this behavior was not replicated by (B) M23-PP / HA NPs.

**Table S1.** Coating M23-PP NPs with HA-based polymers improved their stability (see Supporting information: Stability Table S1.xls), as evidenced by the loss of bactericidal activity after 1 day of incubation at 37 °C, compared to 3 days for M23-PP/HA and M23-PP/HAMS. The MIC was not affected and the MAC increased to 78 nM in M23 after 3 days for the HA-coated formulations. These experiments were run in 5 replicates, N=5.

| **Time (days)** | **Antimicrobial effect** ^[12]^ | | |
| --- | --- | --- | --- |
|  | **M23-PP NPs** | **M23-PP/HA NPs** | **M23-PP/HAMS NPs** |
| 0 | Bactericidal | Bactericidal | Bactericidal |
| 1 | Bactericidal/Activity loss | Bactericidal | Bactericidal |
| 3 | Activity loss | Bactericidal/Activity loss | Bactericidal/Activity loss |
| 7 | Activity loss | Bactericidal/Activity loss | Bactericidal/Activity loss |

**Figure S10.** Biocompatibility of the formulations in the context of bone development was evaluated by assessing the ability of the MC3T3-E1 cell line to undergo osteogenic differentiation and form a calcified bone matrix. ^[13]^ Following treatment with M23-PP/HA, M23-PP/HAMS or PBS for 2 h, the cells were washed with PBS and cultured in white α-MEM supplemented with 50 µg/ml ascorbic acid and 5 mM β-glycerophosphate. Osteogenic differentiation was assessed by AP activity determination at 14 days post-induction (**Figure S10A**) and matrix mineralization on day 21 (**Figure S10B**). ^[13]^ AP activity was quantified colorimetrically after the hydrolysis of p-nitrophenyl phosphate by MC3T3-E1 cells incubated in α-MEM for 2 hours at 37 °C in a 5% CO₂ atmosphere. Absorbance was measured at 405 nm (Tecan Spark microplate reader, Switzerland) and a calibration curve was established using serial dilutions of commercial AP. For studying MC3T3-E1 mineralization, the cells were fixed with 4% paraformaldehyde for 15 minutes at room temperature, washed with PBS, and stained with Alizarin red according to the manufacturer instructions. After washing with deionized water, the cells were imaged with Leica Fluorescence Leica DM IL LED Fluo 11521265 Microscope (LAS X 3.7.6 software). All the experiments were run in 3 independent replicates. The induction of MC3T3-E1 cells following treatment indicates that the formulations support cellular viability and differentiation, demonstrating compatibility with bone-forming cell function. These results, together with those informed in **Figure 8** (Main manuscript), confirm the suitability of M23-PP/HA, M23-PP/HAMS for applications related to bone tissue engineering or bone-related therapeutic strategies.


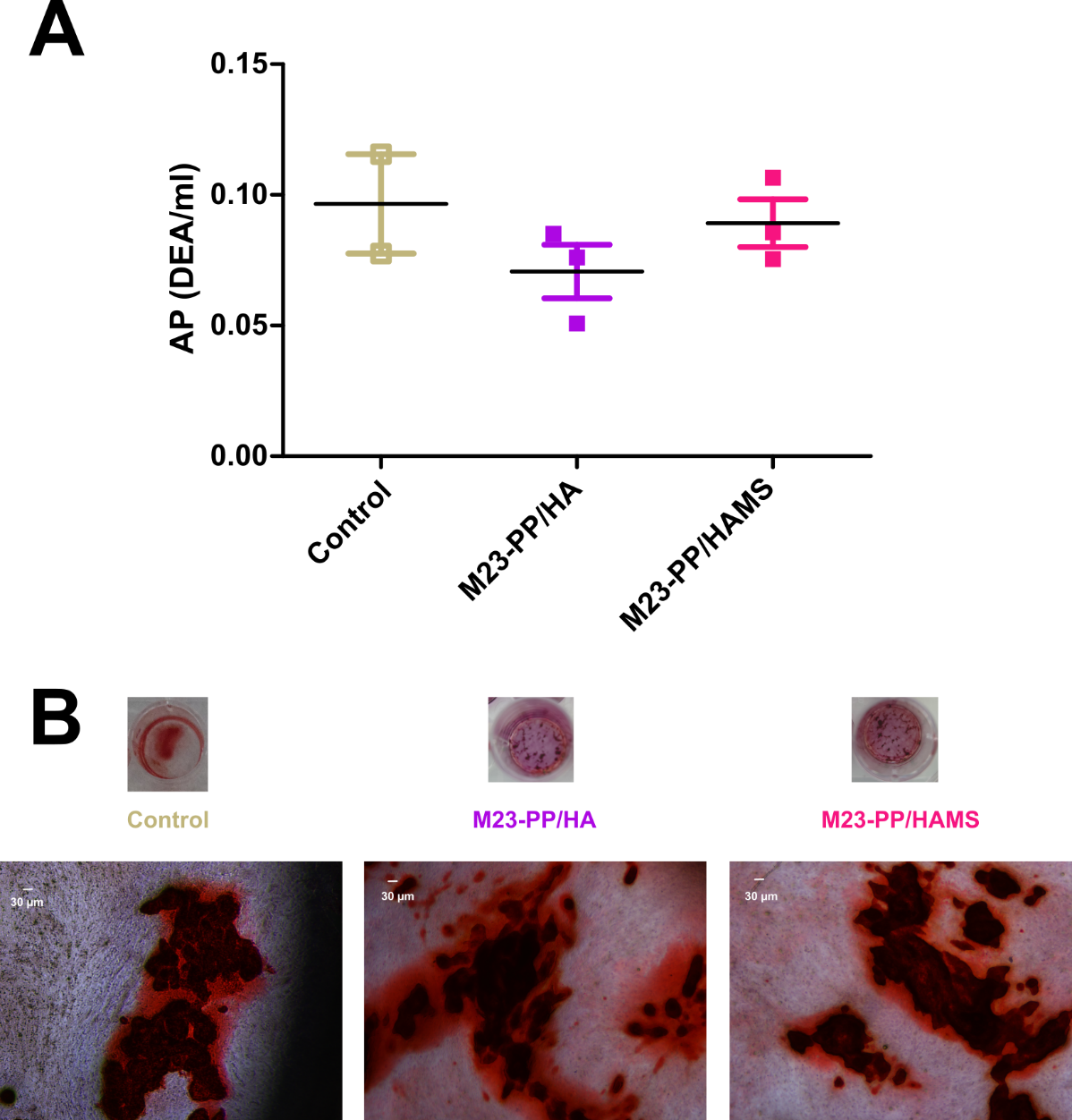


**References**

[1] Reuter Wilhad M., Inc. PerkinElmer, *Application Brief. Liquid Chromatography: The Analysis of a Broad Range of Organic Acids by HPLC with UV Detection* , Waltham, MA 02451 , **2015**.

[2] M. T. Zekarias, G. N. Rao, *Journal of the Chilean Chemical Society* **2012**, *57*, DOI 10.4067/s0717-97072012000100021.

[3] M. Chen, V. Gupta, A. C. Anselmo, J. A. Muraski, S. Mitragotri, *Journal of Controlled Release* **2014**, *173*, DOI 10.1016/j.jconrel.2013.10.007.

[4] G. E. Cheney, Q. Fernando, H. Freiser, *Journal of Physical Chemistry* **1959**, *63*, DOI 10.1021/j150582a018.

[5] C. Leichner, M. Jelkmann, A. Bernkop-Schnürch, *Adv Drug Deliv Rev* **2019**, *151–152*, 191. DOI 10.1016/j.addr.2019.04.007

[6] K. Sunitha, P. Suresh, M. S. Santhosh, M. Hemshekhar, R. M. Thushara, G. K. Marathe, C. Thirunavukkarasu, K. Kemparaju, M. S. Kumar, K. S. Girish, *Int J Biol Macromol* **2013**, *55*, 39. DOI 10.1016/j.ijbiomac.2012.12.047

[7] H. Prinz, *J Chem Biol* **2010**, *3*, 37. DOI 10.1007/s12154-009-0029-3

[8] A. Aitken, M. P. Learmonth, in *Protein Protocols Handbook* (Ed: Walker JM), Humana Press, **2009**, pp. 3–6. DOI 10.1007/978-1-59745-198-7_1

[9] N. Olsen, E. Thiran, T. Hasler, T. Vanzieleghem, G. Belibasakis, J. Mahillon, M. Loessner, M. Schmelcher, *Viruses* **2018**, *10*, 438. DOI 10.3390/v10080438

[10] S. G. Moon, D. Kothari, W. L. Kim, W. Do Lee, K. Il Kim, J. Il Kim, E. J. Kim, S. K. Kim, *J Anim Sci Technol* **2021**, *63*, 1286. DOI 10.5187/jast.2021.e110

[11] L. Drago, L. Cappelletti, E. De Vecchi, L. Pignataro, S. Torretta, R. Mattina, *APMIS* **2014**, *122*, 1013. DOI 10.1111/apm.12254

[12] M. C. Verdi, C. Melian, P. Castellano, G. Vignolo, M. Blanco Massani, *Int J Food Sci Technol* **2020**, *55*, 267. DOI 10.1111/ijfs.14302

[13] A. Semicheva, U. Ersoy, A. Vasilaki, I. Myrtziou, I. Kanakis, *Int J Mol Sci* **2024**, *25*, DOI 10.3390/ijms25084180.

**Thiolated hyaluronic acid: a gateway for targeted killing of *Staphylococcus aureus* on ´the race for surface´ colonization**

**ToC**

Thiolated hyaluronic acid (HAMS) synthesized and characterized by NMR, solubility, thiol content, pKa, is degraded by Staphylococcal hyaluronate lyase but not by mammalian hyaluronidase.

Coating polyphosphate–M23 phage endolysin nanoparticles (M23-PP) with HAMS confers *Staphylococcus aureus* responsiveness. M23-PP/HAMS are studied for size, charge, morphology, release profile, safety, and efficacy.

M23-PP/HAMS provide a switch-response to avoid *S. aureus* implant-related infection.


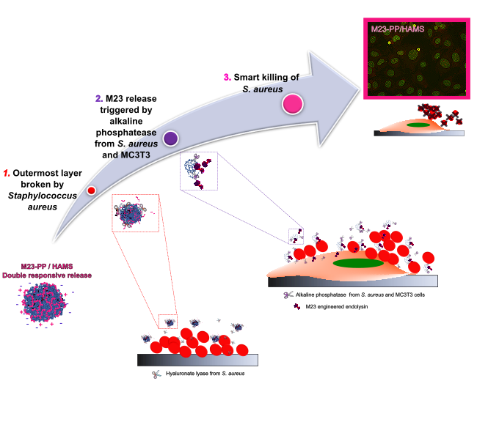

Supplement: Supplementary file 1 — Supporting Information [file ADHM-15-0-s001.docx]
